# Surfactants Enhanced Heavy Oil–Solid Separation from Carbonate Asphalt Rocks-Experiment and Molecular Dynamic Simulation

**DOI:** 10.3390/nano11071835

**Published:** 2021-07-14

**Authors:** Jinjian Hou, Jinze Du, Hong Sui, Lingyu Sun

**Affiliations:** 1 School of Chemical Engineering and Technology, Tianjin University, Tianjin 300072, China; houjinjian@tju.edu.cn (J.H.); sunlingyu4321@163.com (L.S.); 2 National Engineering Research Centre of Distillation Technology, Tianjin 300072, China; 3 Collaborative Innovation Center of Chemical Science and Engineering, Tianjin 300072, China

**Keywords:** surfactants, carbonate asphalt rocks, molecular dynamic simulation, oil–solid separation

## Abstract

In this study, surfactants were used to enhance heavy oil–solid separation, and a detailed mechanism was explored by SARA (saturates, aromatics, resins, asphaltenes) analysis, element analysis, AFM measurement, and molecular dynamic simulation. Surfactants could effectively decrease oil/solid interaction force and then oil–solid separation would be enhanced. The oil–solid interactive force was in relation to surfactants concentration, pH value, asphaltene content, and salinity. The molecular dynamics simulation results show that the dissociation of saturated hydrocarbon, aromatic hydrocarbon, resin, and asphaltene (SARA) on carbonate minerals is gradually weakened for all surfactants. In the process of molecular dynamics simulation of surfactant stripping SARA, firstly, the surfactant molecules adsorb on the surface of SARA molecules. After that, the surfactant peels SARA molecules off the surface of calcite under the influence of molecular thermal motion. In this process, surfactant molecules will not be directly adsorbed on the surface of trace minerals. The results of energy/temperature balance indicated that saturates, aromatics and resins could remain stable when the molecular dynamics simulation time reached 2000 ps with the phenomenon that saturates, aromatics could liberate from minerals totally within 2000 ps. The molecular dynamics simulation of asphaltenes will not liberate from calcite surface within 6000 ps, meanwhile, they could not reach the energy balance/energy balance within 6000 ps. The functional groups of surfactant molecules would have interactions with the SARA functional group, resulting in different dissociation effects of SARA. The results of molecular dynamics simulation are consistent with the experiment results. The separation effect of saturated hydrocarbon, aromatic hydrocarbon, resin, and asphaltene in five kinds of surfactants were different. The molecular dynamic simulation results were in accordance with the SARA analysis.

## 1. Introduction

Enhanced oil recovery (EOR) was the process that extracting residual oil after primary/secondary oil recovery process [1]. EOR mainly included three methods: miscible enhanced oil recovery (M-EOR) [2,3], thermal enhanced oil recovery (T-EOR) [4,5], and chemical enhanced oil recovery(C-EOR) [6,7]. C-EOR has been industrialized, which showed unique properties than other methods. Surfactants [8,9], polymers [10,11], alkalis [12,13], ions [14,15], foams [16,17], and nanofluids [18,19,20,21,22] and other chemicals were used during the C-EOR process. The mechanism of EOR process was wettability alteration [23,24], interfacial tension decrease [25,26,27], structural disjoining pressure alteration [28,29], viscosity decrease [30], and other mechanisms [21]. Among these mechanisms, the oil–solid interaction force was the main mechanism of oil recovery. When the oil–solid interaction force decreased, the oil recovery would increase.

Carbonate asphalt rocks are widely distributed, and the reserves are huge. Therefore, carbonate asphalt rocks have attracted many researchers’ attention. Different methods have been used to recover bitumen from carbonate asphalt rocks. Hot-water-based extraction (HWBE) has been industrialized, but the method is only suitable for the water-wet asphalt rocks, such as the Canadian oil sands [31]. Due to the fact that the interaction force between calcium carbonate and heavy oil is strong, the bitumen recovery rate is low from carbonate asphalt rocks by HWBE process [32]. In order to overcome the HWBW disadvantages, solvent extraction, pyrolysis and other methods have been put forward. Solvent extraction can obtain high heavy oil recovery, but there are some disadvantages [33], the organic solvents were toxicity, flammable, solvent loss, and residual solvent in minerals, and other problems [34,35]. Pyrolysis could recover the light oils, and the heavy oil components could be decomposed under high temperature. However, the pyrolysis consumed high amounts of energy and there were fine particles in oil, which limited the pyrolysis application.

Hou et al. [36] studied that bitumen covered the surface of carbonate minerals surface, some calcium carbonate exposed to the surroundings. Therefore, the reaction extraction method was put forward to extract bitumen from carbonate asphalt rocks. Namely, the carbonate asphalt rocks were dissolved into acid solutions, and most CaCO_3_ reacted with acid, and then bitumen would be recovered. Li et al. [37] used the formic acid to reacted with carbonate asphalt rocks. However, due to the high cost of formic acid, Hou et al. [36] used hydrochloric acid to do the reaction extraction process.

Although hydrochloric acid could achieve heavy oil recovery from carbonate asphalt rocks, the bitumen recovery and B/S was low, which limited the industrial application of reaction extraction process. The reason why the bitumen recovery and B/S was low was that the interaction force between CaCO_3_ and bitumen was high. Therefore, it is necessary to find novel methods to decrease oil–solid interaction force.

Based on the above background, we used surfactants to enhance reaction extraction process for carbonate asphalt rocks. The reasons were as follows: (1) Surfactants could be divided into chemical surfactants and bio-surfactants, they could effectively decrease heavy oil–solid interaction force [38,39]; (2) Surfactants are the most common chemicals in the EOR process [40]. He et al. [41] used the nonionic surfactant/nanoparticle composite system to enhanced oil recovery, the oil recovery rate increased by 16.8%. Zhan et al. [42] used the low concentration of alkylaryl sulfonate surfactant to enhanced oil recovery and the main mechanism was the solubilization of surfactants. Machale et al. [43] studied the natural surfactant effect on oil/water interfacial tension, and the surfactant was used to enhanced oil recovery. However, studies on surfactants enhanced reaction extraction for carbonate asphalt rocks are few.

Although surfactants have been widely used in the EOR process, the mechanisms of surfactants’ enhanced oil recovery are unclear. On the one hand, some researches indicated that surfactants could adsorb onto minerals surface, and they occupy the heavy oil components adsorption sites, and then heavy oil components would liberate from minerals surface [44]. On the other hand, some studies have indicated that surfactant molecules could adsorb the oil components surface, and then the surfactant molecules would “tow” heavy oil components liberation from minerals surface [45,46]. In this study, we used the molecular dynamics to explore the surfactants’ behavior on the oil–solid interface and oil-water interface, which could explain the enhancement mechanism.

In this study, we used surfactants to enhance bitumen recovery from carbonate asphalt rocks by hydrochloric acid-reaction extraction method, and study the surfactant mechanism. The purpose of this study was to: (i) explore different surfactants effect on bitumen recovery and B/S of the reaction extraction method; (ii) explore surfactants species, concentration, pH, salinity and asphaltene content effect on the oil–solid interaction force; (iii) use molecular dynamics simulation to study surfactants’ behavior on oil/water interface and oil/solid interface, and surfactant functional groups were studied.

## 2. Materials and Methods

### 2.1. Materials

Sulphuric acid (H_2_SO_4_(98%)), hydrogen peroxide(H_2_O_2_(30%)), alcohol(C_2_H_5_OH), sophorolipid(C_32_H_58_O_13_), rhamnolipid (C_16_H_30_O_7_), cetyltrimethylammonium bromide (CTAB,C_19_H_42_BrN), sodium dodecyl sulfate (SDS, C_12_H_25_SO_4_Na), Triton X-100 (TX-100, C_18_H_30_O_3_), hydrochloric acid (HCl), trichloroethylene(C_2_HCl_3_), methanol(CH_3_OH), cyclohexane(C_6_H_12_), toluene(C_7_H_8_), and n-heptane(C_7_H_16_) were analytically pure from Tianjin Jiangtian Technology Co. Ltd., Tianjin, China. Carbonate asphalt rocks were lipophilicity from Buton, Indonesian. AFM probe (SNL 10) was from Brucker, Karlsruhe, Germany. Calcium carbonate (CaCO_3_), sodium hydroxide (NaOH), gamma-aluminium oxide (γ-Al_2_O_3_), and high purity water (H_2_O, 18MΩ) were from Tianjin Jiangtian Technology Co. Ltd., Tianjin, China. Single-crystal silicon (Si), silicon dioxide (SiO_2_) microspheres and PTEF were from Zhongjingkeji Technology Co. Ltd., Shanghai, China.

### 2.2. Surfactants Enhanced Heavy Oil–Solid Separation with Acid Solution Aid

**Figure 1:**
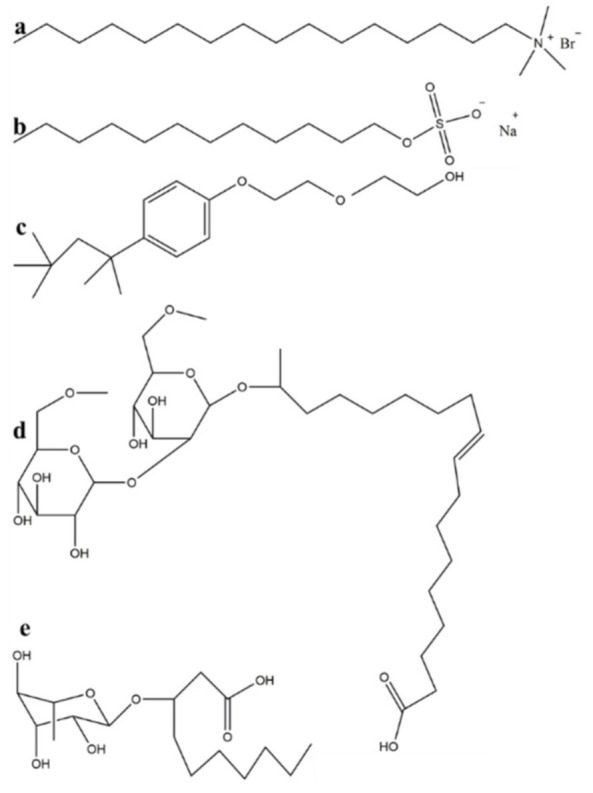
Structure of five surfactants (**a**) CTAB; (**b**) SDS; (**c**) TX-100; (**d**) sophorolipid; (**e**) rhamnolipid.

The main composition of carbonate asphalt rocks was CaCO_3_. Carbonate asphalt rocks could react with HCl to produce CO_2_, CaCl_2_ and bitumen, and the process was called reaction extraction (acid cleaning) [36,37]. In this study, surfactants were used to enhance the reaction extraction process. The structure of five surfactants (CTAB, SDS, TX-100, sophorolipid, rhamnolipid) were shown in Figure 1. Five surfactants (CTAB, SDS, TX-100, sophorolipid, rhamnolipid) at 500 ppm were dispersed into 7.4 wt % HCl solution, which formed surfactant-HCl complex solution. 50 g carbonate asphalt rocks were mixed with 200 mL surfactant-HCl complex solution. The reaction extraction process conditions were as follows: stirring rate was 600 rpm, temperature was 60 °C, and reaction time was 4 h. The condensing tube was used to cool the volatile the HCl, in order avoid the HCl volatilize. The generated gas could be was exhausted by the air pipe. During the reaction extraction process, the bitumen could float onto the solution surface, and form the bitumen froth.

### 2.3. Heavy Oil Product Analysis

#### 2.3.1. Heavy Oil Recovery and B/S Analysis

After the reaction extraction process, bitumen could be recovered. The bitumen recovery and B/S were calculated by the Equations (1) and (2), R represents the oil recovery, m_f_ represents the recovered heavy oil, m_o_ represents the total mass of heavy oil, m_s_ represents the carbonate minerals mass.

R = m_f_/m_o_ (1)

B/S = m_f_/m_s_ (2)

#### 2.3.2. SARA Analysis

Heavy oil could be divided into SARA components (saturates, aromatics, resins, asphaltenes). In this study, the heavy oil by surfactants enhanced reactive extraction was done the SARA analysis. The four fractions were carried out using ASTM D4124. Asphaltenes could dissolve into toluene, but cannot dissolve into n-heptane. The detailed experiment procedure could refer to [47].

#### 2.3.3. Element Analysis

The heavy oil from different surfactants extraction was analyzed, and the elements content was used to value the surfactants’ effect. 

### 2.4. Surfactant Enhanced Oil Recovery Mechanism

#### 2.4.1. Oil–Solid Interaction Force Analysis

The interactive force between heavy oil and carbonate minerals varies widely. In the process of oil–solid separation, the interaction between heavy oil and solid affects the difficulty of oil–solid separation. Atomic force microscopy (AFM) (Brucker, Karlsruhe, Germany) can effectively measure the strength of the interaction between heavy oil and mineral surface. When the probe is close to the mineral surface, there is repulsive force between them; while when the probe is away from the mineral surface, there is an attractive force between them, which is adhesive force. Adhesive force is a measure of the interaction between heavy oil and minerals. When the adhesion force decreases, the heavy oil was easier to liberate from minerals. 

The heavy oil was extracted from carbonate asphalt rocks by Soxhlet extraction. A 1 g measure heavy oil was dissolved into 100 mL toluene to form 1 wt % bitumen solution, and then Then, 1 wt % asphalt toluene solution was diluted to 0.02 wt % asphalt toluene solution for subsequent use.

The SiO_2_ microspheres were washed with acetone, ethanol, and distilled water. Then, the SiO_2_ spheres were dried in a vacuum dryer, and trimethoxysilane was put into the bottom of the dryer. The drying time was 24 h, so that C_8_ was grafted on the SiO_2_ surface, and then SiO_2_ microspheres became hydrophobic. Then, it was washed with toluene and ethanol and dried with nitrogen.

**Figure 2:**
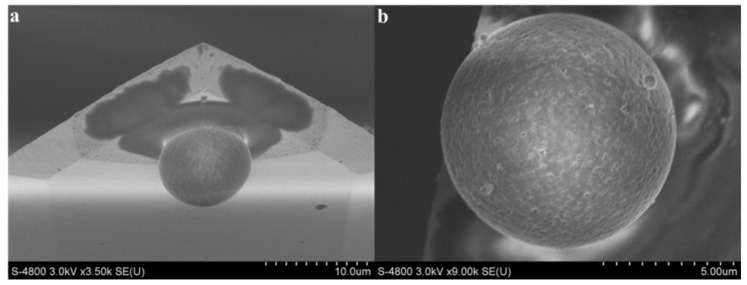
Morphology of SiO_2_ sphere covered with heavy oil (**a**) vertical view; (**b**) front view.

The modified SiO_2_ microspheres were placed in 0.02 wt % bitumen toluene solution and vibrated to make the surface of SiO_2_ spheres absorb bitumen. After 4 h, the solution was dropped onto the glass slide. The AB adhesive was mixed evenly according to the proportion, the probe was moved to the edge of the mixed AB adhesive, and then needle was fed in slowly, so that there is a small amount of AB adhesive on the tip of the needle. Then, put the probe close to the SiO_2_ microsphere area stained with asphalt on the glass slide, and slowly enter the needle to make the asphalt ball stick to the tip of the needle. The element analysis of SiO_2_ sphere covered with heavy oil was shown in Table 1, which indicated the SiO_2_ spheres adhered to the tip of the needle. The morphology of SiO_2_ sphere covered with heavy oil was shown in Figure 2. 

Calcite with smooth and clean surface was selected and washed with acetone, ethanol, and distilled water. At the same time, double-sided adhesive was used to stick the surface of calcite on the stainless steel sheet, so as to carry out the follow-up experiment. Clean the calcite with 2% hellmanex solution, then wash with distilled water and blow dry with nitrogen. Then, AFM was used to measure the force curves between bitumen and calcium carbonate in different surfactant solutions. Five kinds of surfactants (CTAB, SDS, TX-100, rhamnolipid, sophorolipid) were tested. At the same time, the adhesion force alterations with surfactant concentration, pH, asphaltenes content, and salt ion concentration were tested. 

#### 2.4.2. Molecular Dynamics Simulation

**Figure 3:**
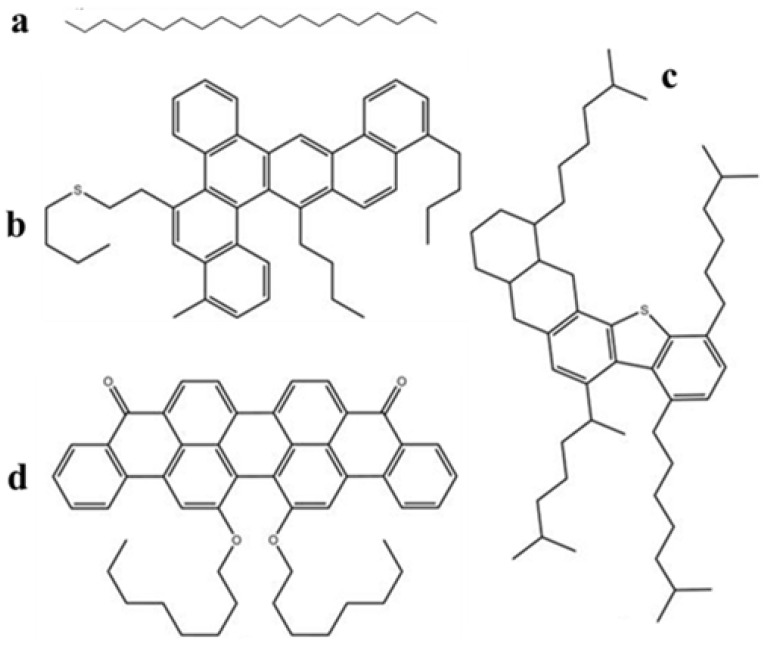
Molecular structures of SARA fractions: (**a**) saturates (C_20_H_42_); (**b**) aromatics (C_46_H_50_S); (**c**) resins (C_50_H_80_S); (**d**) asphaltenes (C_50_H_48_O_4_).

In order to explore the surfactants role on heavy oil–solid separation, molecular dynamics simulation was done, and Materials Studio (MS) software was the software to do the molecular dynamic simulation process. Molecular structures of SARA fractions were shown in Figure 3. The process could be divided into three procedures: (1) SARA adsorbed onto the calcite surface; (2) five surfactant–water solutions were added into the systems; (3) heavy oil liberated from minerals. The detailed molecule dynamic simulation was shown as follows.

(1) The (1 0 4) plane of calcite crystal was chosen as the calcium carbonate surface. In order to decrease the system energy, the energy and structure optimization were carried out. The supercell was built, and the vacuum layer was 50 Å, and then the periodicity became three-dimensional. The cell parameters were a = 72.86228 Å, b = 29.940008 Å, c = 115.916234 Å. Interfacial angle was α = 90°, β = 90°, γ = 90°.
(2) Put saturates (optimized) molecules into the amorphous cell, then run the COMPASS field of force.
(3) Build layers, layer 1 was calcite surface, layer 2 was saturates unit cell, and saturates were used to adsorb onto the calcite surface, and the adsorption time continued 1000 ps.
(4) Do the surfactants solution enhanced heavy oil liberation process, namely, build the unit cell filled with 20 surfactant molecules and 1000 water molecules, and then build the corresponding layer structure. Saturates would desorb from minerals.
(5) The simulation process conditions were as follows: run module was Forcite, NVT ensemble, compass force field, 298 K, Berendsen thermostat. Simulation time was 2000 ps, the step was 1fs. Adsorption process and desorption process followed simulation condition.

To summarize, the SARA adsorption simulation and desorption simulation used the Forecite field of force, and classical dynamics model.

The task of the molecular dynamic simulation was to study the SARA total liberation time, relative concentration, mean square displacement in five surfactants solutions, and the energy balance and energy balance of the SARA components in five surfactants solutions were studied. In the end, the surfactant functional groups’ role on SARA liberation was studied. 

## 3. Results and Discussion

### 3.1. Surfactants Enhanced Heavy Oil–Solid Separation

#### 3.1.1. Heavy Oil Recovery and B/S Analysis

**Figure 4:**
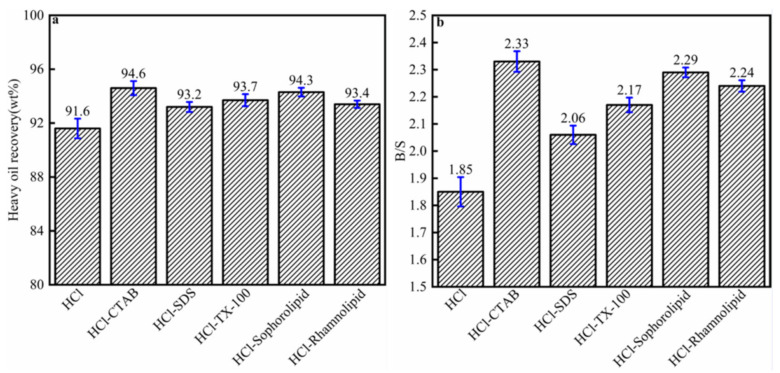
(**a**) Recovery; (**b**) B/S of heavy oil from different surfactants enhanced carbonate oil–solid separation.

The heavy oil recovery and B/S of different surfactants were shown in Figure 4. The heavy oil recovery was as follows (Figure 4a): CTAB (94.6 wt %) > sophorolipid (94.3 wt %) > TX-100 (93.7 wt %) > rhamnolipid (93.4 wt %) > SDS (93.2 wt %) > without surfactants (91.6 wt %). The results indicated that surfactants could efficiently enhance the heavy oil–solid separation from carbonate asphalt rocks. The B/S of the extracted heavy oil was as follows (Figure 4b): CTAB (2.33) > sophorolipid (2.29) > rhamnolipid (2.24) > TX-100 (2.17) > SDS (2.06)> without surfactants (1.85). Figure 4 showed that surfactants could enhance the reaction extraction process, which increased heavy oil recovery and B/S.

#### 3.1.2. SARA Analysis

The SARA analysis of the heavy oil from Soxhlet extraction, HCl direct reaction extraction and different surfactants enhanced-HCl reaction extraction was shown in Table 2. The SARA content of heavy oil from Soxhlet extraction was as follows: saturates (18.07 wt %), aromatics (31.28 wt %), resins (28.32 wt %), and asphaltenes (22.33 wt %). The SARA contents of heavy oil by HCl directly reactive extraction was as follows: saturates (17.78 wt %), aromatics (20.69 wt %), resins (28.89 wt %), and asphaltenes (22.64 wt %). The SARA contents from different surfactants enhanced-HCl reaction extraction showed huge differences. The saturates content was as follows: CTAB (17.21 wt %) > sophorolipid (17.14 wt %) > TX-100 (16.89 wt %) > SDS (16.58 wt %) > rhamnolipid (16.34 wt %). The aromatics content was as follows: sophorolipid (30.04 wt %) > TX-100 (29.86 wt %) > SDS (29.71 wt %) > rhamnolipid (29.56 wt %) > CTAB (29.12 wt %). The resins content was as follows: rhamnolipid (30.12 wt %) > sophorolipid (29.37 wt %) > SDS (29.33 wt %) > TX-100 (29.25 wt %) > CTAB (29.19 wt %). The asphaltenes content was as follows: CTAB (24.48 wt %) > SDS (24.38 wt %) > TX-100 (24 wt %) > sophorolipid (23.45 wt %) > rhamnolipid (23.98 wt %). When surfactants were used to enhance-HCl reaction extraction, the heavy components (resins/asphaltenes) content would increase, and light components (saturates/aromatics) content would decrease, which indicate that surfactants would help the heavy component recovery. 

#### 3.1.3. Element Analysis

Element analysis of seven different bitumen samples by different surfactant enhanced methods was shown in Table 3. The element content of the heavy oil from Soxhlet extraction was C (80.795 wt %), H (9.527 wt %), other elements (9.678 wt %), and C/H was 8.48. The element content of the heavy oil by HCl directly reactive extraction was as follows: C (81.242 wt %), H (9.423 wt %), other elements (9.335 wt %), and C/H was 8.62. The reactive extraction process could increase heavy oil recovery and heavy components content, therefore, C element relative content would increase. When surfactants were used to enhance HCl reaction extraction process, the C element content of heavy oil would increase, H element would decrease, therefore, C/H would increase. The C/H of the heavy oil extracted by different surfactants enhanced HCl reaction extraction was as follows: CTAB (9.13) > sophorolipid (9.02) > TX-100 (8.94)> rhamnolipid (8.88) > SDS (8.82) > without surfactants (8.62). 

### 3.2. Oil–Solid Interaction Force Analysis 

#### 3.2.1. Surfactants Concentration 

**Figure 5:**
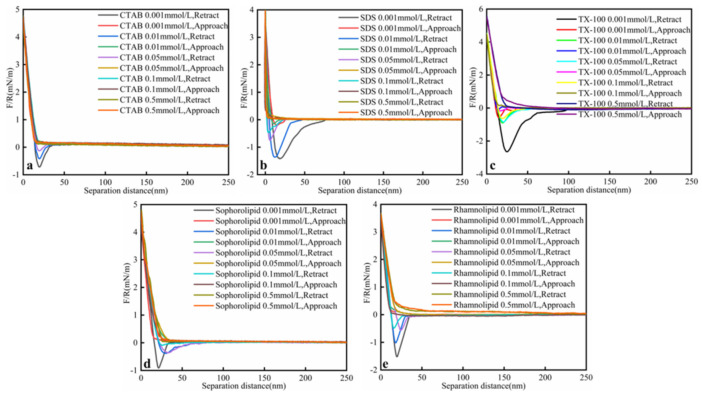
Interaction forces (F/R) between heavy oil and calcium carbonate in different surfactants concentrations (**a**) CTAB; (**b**) SDS; (**c**) TX-100; (**d**) Sophorolipid; (**e**) Rhamnolipid solutions as a function of the separation distance at ambient conditions (25 °C, 1 mmol/L KCl, PH = 3.5, 45 ppm asphaltenes).

Interaction forces (F/R) between bitumen and calcium carbonate in different concentrations of surfactants solution as a function of separation distance was shown in Figure 5. Five surfactants could effectively decrease heavy oil–solid adhesive force (Table 4). When the CTAB concentration was 0.001 mmol/L, the oil–solid adhesive force was 0.72 mN/m. When CTAB concentration increased, the oil–solid adhesive force decreased. The SDS decrease oil–solid adhesive force effect was weaker than CTAB. When SDS concentration was 0.001 mmol/L, the oil–solid adhesive force was 1.41 mN/m. When SDS concentration increased to 0.5 mmol/L, the oil–solid adhesive force decreased to 0. When TX-100 concentration was 0.001 mmol/L, the adhesive force was 2.64 mN/m, which means that the TX-100 effect on oil–solid adhesive force was weak on low concentration. Biosurfactants (sophorolipid, rhamnolipid) could effectively decrease oil–solid adhesive force. When sophorolipid and rhamnolipid concentration was 0.1mmol/L, the oil–solid adhesive force was 0.1 mN/m, 0.48 mN/m, respectively. The oil–solid adhesive force decreased when the surfactants concentration increased. When the surfactants’ concentration was 0.5 mmol/L, the oil–solid adhesive force decreased to 0 (TX-100 except). When the surfactants’ concentration was 0.001 mmol/L, the adhesive force was as follows: CTAB <sophorolipid < SDS < rhamnolipid < TX-100; When the surfactants’ concentration was 0.05 mmol/L, the adhesive force was as follows: CTAB < sophorolipid < rhamnolipid < SDS < TX-100. The oil–solid adhesive force altered when the surfactants’ concentration altered. When the oil–solid adhesive force decreased, the heavy oil recovery and B/S increased. 

#### 3.2.2. PH

**Figure 6:**
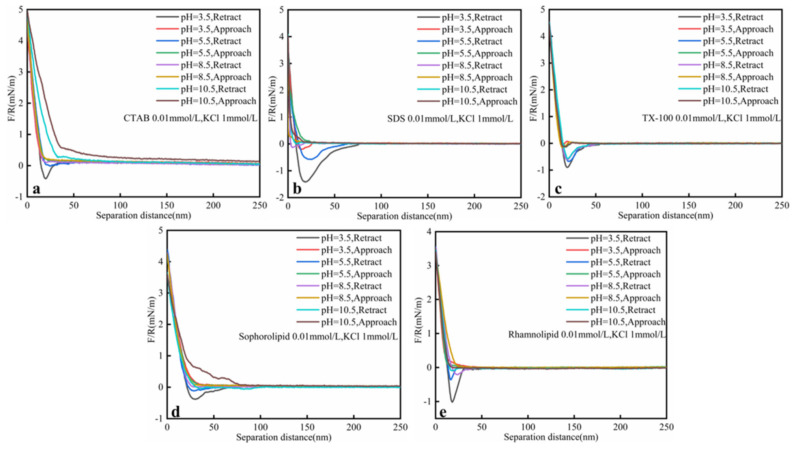
Interaction forces (F/R) between heavy oil and calcium carbonate at different pH surfactants (**a**) CTAB; (**b**) SDS; (**c**) TX-100; (**d**) Sophorolipid; (**e**) Rhamnolipid solutions as a function of the separation distance at ambient conditions (0.01 mmol/L surfactants, 25 °C, 1 mmol/L KCl, 45 ppm asphaltenes).

As shown in the Figure 6 and Table 5, heavy oil–solid interaction force decreased with higher pH. The surfactants’ adsorption onto oil–solid interface would increase when pH increased. Hot-water-based extraction (HWBE) was done at higher pH due to the lower oil–solid interaction force. 0.01 mmol/L surfactants make the oil–solid adhesive force followed the rules: CTAB < sophorolipid<rhamnolipid < TX-100 < SDS (at higher pH, SDS < TX-100). When the pH was 10.5, CTAB, SDS and sophorolipid decreased the oil–solid adhesive force to 0.

#### 3.2.3. Asphaltenes Content

**Figure 7:**
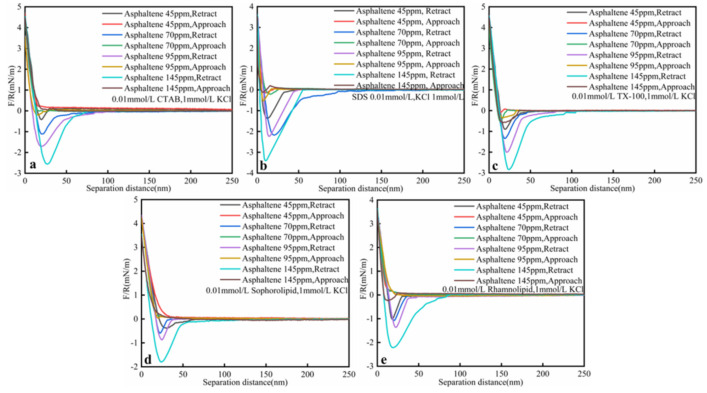
Interaction forces (F/R) between heavy oil and calcium carbonate at different asphaltenes concentration (**a**) CTAB; (**b**) SDS; (**c**) TX-100; (**d**) Sophorolipid; (**e**) Rhamnolipid solutions as a function of the separation distance at ambient conditions (0.01 mmol/L surfactants, 25 °C, 1 mmol/L KCl, pH = 3.5).

As was shown in Figure 7 and Table 6, oil–solid interaction force increased with higher asphaltenes concentration. Asphaltenes are the most complex component among SARA. When the asphaltenes content increased, the heavy oil viscosity increased, and then oil–solid separation became more difficult. When asphaltene concentration was 45 ppm, the oil–solid adhesive force followed the rule: sophorolipid (0.39 mN/m) < CTAB (0.42 mN/m) < TX-100 (0.89 mN/m) < rhamnolipid (1.02 mN/m) < SDS (1.36 mN/m). When asphaltene concentration increased to 145 ppm, the oil–solid adhesive force followed the rule: sophorolipid (1.80 mN/m) < rhamnolipid (2.21 mN/m) < CTAB (2.56 mN/m) < TX-100 (2.84 mN/m) < SDS (3.39 mN/m). At low asphaltenes concentration, the rhamnolipid effect was not so obvious. When the asphaltenes concentration increased, the rhamnolipid could effectively decreased the oil–solid interaction force. In other words, rhamnolipid showed optimal effect in decreasing oil/solid interaction force at higher asphaltenes concentrations. 

#### 3.2.4. Salinity 

**Figure 8:**
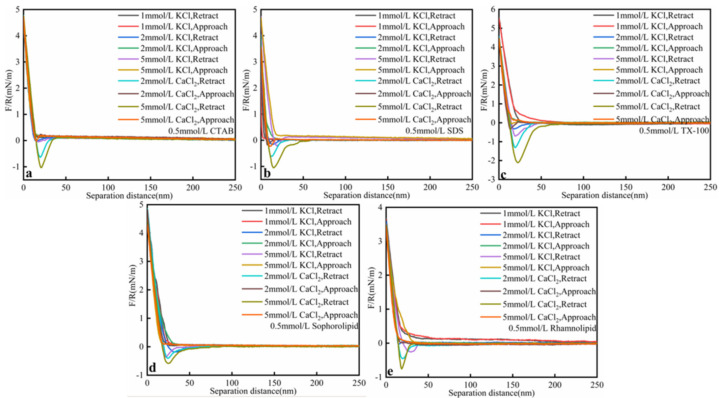
Interaction forces (F/R) between heavy oil and calcium carbonate at different salinities (**a**) CTAB; (**b**) SDS; (**c**) TX-100; (**d**) Sophorolipid; (**e**) Rhamnolipid solutions as a function of the separation distance at ambient conditions (0.01 mmol/L surfactants, 25 °C, pH = 3.5, 45 ppm asphaltenes).

As was shown in Figure 8 and Table 7, salinity would influence oil–solid interaction force in surfactant solutions. When salinities increased, oil–solid interaction force increased. At the same salinity, Ca^2+^ could make oil–solid interaction force higher than K^+^, which was due to the fact that Ca^2+^ could help oil–solid form bridge connection effect. For instance, when CaCl_2_ concentration was 5 mmol/L, the oil–solid interaction force for CTAB, SDS, TX-100, sophorolipid, rhamnolipid was 1.03, 1.05, 2.10, 0.58, and 0.76 mN/m, respectively. However, when KCl concentration was 5 mmol/L, the oil–solid interaction force for CTAB, SDS, TX-100, sophorolipid, rhamnolipid was 0.04, 0.22, 0.70, 0.36, and 0.26 mN/m, respectively. Compared to chemical surfactants (CTAB, SDS, TX-100), biosurfactants (sophorolipid, rhamnolipid) showed higher salt resistance. When CaCl_2_ concentration reached 2 mmol/L, the oil–solid interaction force in biosurfactants was lower than that in chemical surfactants. In industrial production, biosurfactants should be chosen in industrial saline.

### 3.3. Molecular Dynamics Simulation

#### 3.3.1. Saturates–Solid Separation Process

##### Saturates Liberation Conformation Analysis

When saturates adsorbed onto calcite surface for 1 ns, saturates adsorption would become stable. Then the aqueous surfactants solutions were added into the simulation system. Saturates would desorb from calcite surface in different surfactant solutions. The complete liberation time was different in different surfactant solutions. When heavy oil components completely liberated from minerals surface, the heavy oil components and minerals surface would be filled with H_2_O molecules. When the total liberation time decreased, the heavy oil components were easy to liberate from minerals’ surfaces. 

Saturates could liberate from calcite surface in five surfactants solutions, as was shown in Figure S1. Because saturates were light components, and the interaction force between saturates and calcite surface was low, saturates were easy to liberate from calcite surface. Chemical surfactants and biosurfactants enhanced oil recovery process was similar. CTAB, SDS, TX-100, and rhamnolipid molecules connected with saturate molecules, and then these surfactant molecules were near to the saturate molecules’ surface. The surfactant molecules dragged saturate molecules, liberating them from the minerals’ surfaces. Saturates were easy to concentrate and aggregate, and then saturates liberated from calcite surface. The saturates were far away from calcite surface, and permeated into the surfactant solutions. The process that saturates liberated from calcite surface in biosurfactants sophorolipid was different from other surfactants. Saturates were divided into two parts, and the reason was due to the fact that sophorolipid molecules steric hindrance was huge and the interaction force between sophorolipid molecules and saturates was high. After saturates were divided into two parts, sophorolipids molecules would be easy to cover saturates molecules surface, increase the interaction area, which would help saturates to liberate from calcite surface. 

**Figure 9:**
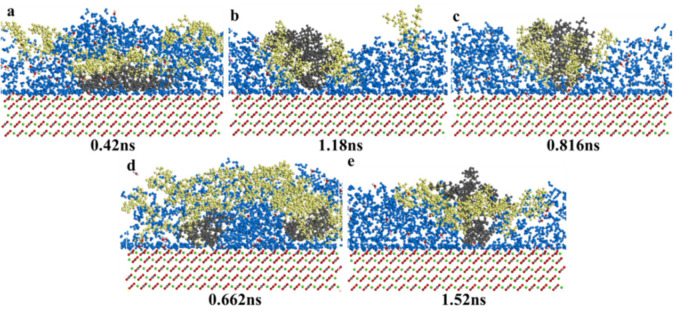
Consecutive snapshot of desorption of saturates from a modeled calcite surface immersed in (**a**) CTAB; (**b**) SDS; (**c**) TX-100; (**d**) Sophorolipid; (**e**) Rhamnolipid-water system just right. Blue = water; black = saturates; red = calcite surface; yellow = surfactants.

Figure 9 showed that the total liberation time of five surfactants was as follows: CTAB (0.42 ns) < sophorolipid (0.662 ns) < TX-100 (0.816 ns) < SDS (1.18 ns) < rhamnolipid (1.52 ns). CTAB showed the strongest interaction with saturates molecules because of the shortest total liberation time. For the biosurfactants, sophorolipid molecules role was obviously stronger than rhamnolipid. 

##### Saturate Concentration Distribution and MSD 

**Figure 10:**
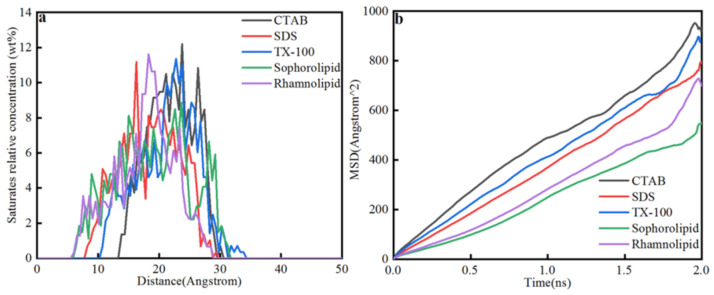
(**a**) Saturates relative concentration; (**b**) Mean square displacement.

Figure 10 showed saturate relative concentration and mean square displacement. Saturate displacement from calcite surface was as follows: CTAB (13.5 Å) > TX-100(10 Å) > SDS (8 Å) > Sophorolipid (6 Å) ≥ Rhamnolipid (6 Å). For chemical surfactants, saturates in CTAB and TX-100 surfactants were mainly concentrated on the calcite surface 15–30 Å displacement, and the saturates in SDS solutions distributed broadly, the displacement was 8–30 Å. For the two biosurfactants, saturates in sophorolipid solutions were mainly distributed on the 6–32 Å, and saturates in rhamnolipid solutions were mainly distributed on the 6–32 Å. Saturates in CTAB solutions displacement was huge, which means that CTAB makes oil phase far away with the calcite surface, and the CTAB surfactants liberation effect was optimal. Saturates in five surfactants solutions the average displacement corresponds with the total liberation time, namely, CTAB (0.42 ns) < sophorolipid (0.662 ns) < TX-100(0.816 ns) < SDS (1.18 ns) < Rhamnolipid (1.52 ns). When the total liberation time was less, then the saturates displacement would be larger, and the saturates main body would be far away from the calcite surface, which means that CTAB showed optimal effect on saturates liberation from calcite surface. As was shown in Figure 10b, the MSD coefficients were as follows: CTAB > TX-100 > SDS > rhamnolipid > sophorolipid. The diffusion coefficient was different from the process of saturate liberation from mineral surfaces. Because the coefficient calculation was different from conformation analysis, because MSD coefficient was not focused on the coefficient.

##### Saturates Energy/Temperature Balance

The change of saturates’ surfactant solutions system energy and temperature with time was shown in Figure S2. The red line is the kinetic energy line, and the kinetic energy remained stable with the time change. Non-bond energy, potential energy, and total energy remained stable. Saturates in CTAB, TX-100, sophorolipid solutions, when the molecular dynamic simulation time reached 1 ns, the simulation systems reached stable. For SDS solutions and rhamnolipid solutions, when the molecular dynamic simulation reached 1 ns, the system did not stabilize, and non-bond energy, potential energy, and total energy decreased with time, due to the fact that the system did not reach equilibrium, and the saturates liberation process proceeded. When the simulation time reached 2 ns, saturates in SDS solutions and rhamnolipid solutions reached equilibrium, and the non-bond energy, potential energy, and total energy reached stability. Temperature was stable when saturates were in CTAB, SDS, TX-100, sophorolipid, and rhamnolipid solutions, the temperature settled in 298K, and the temperature range was within 10 K. Figure 3, Figure 4 and Figure 5 showed that when the simulation time reached 2 ns, the saturates in five surfactant solutions reached energy balance and temperature balance. 

#### 3.3.2. Aromatics–Solid Separation Process

##### Aromatics Liberation Conformation Analysis

Aromatics could liberate from mineral surfaces in five surfactants solutions, as was shown in Figure S3. In Figure S3a–e, all surfactant molecules could promote aromatics liberation from calcite surface, and the process was similar to the saturates process. Surfactant molecules could adsorb onto aromatic molecule surfaces, and then under the action of molecules dynamics, the surfactant molecules would drag aromatic molecules, liberating them from the calcite surface, and all surfactant molecules would not interact with aromatic molecules. Aromatic molecules would concentrate and aggregate, and then when aromatics were completely liberated from calcite surface, the surfactant molecules would interact with aromatics and promote aromatics molecules far away from the calcite surface, and then aromatic molecules would diffuse into surfactant solutions. 

The adsorption behavior between biosurfactants and chemical surfactants on aromatic molecules liberation from calcite surface was different. When aromatics were in sophorolipid solutions, the sopholipid molecules would be in the center to adsorb aromatics, and the sophorolipid molecules would not cover the total aromatics molecules, and due to the fact that sophorolipid molecules showed high steric hindrance, and many sophorolipid molecules adsorbed each other, and some sophorolipid molecules were in aromatics molecules on one side. During the process of rhamnolipid enhanced heavy oil–solid separation, some rhamnolipid molecules were dispersed into the aqueous phase, and they did not interact with aromatics. The reasons were as follows: rhamnolipids molecules have unique molecular structure, and the oxygen-containing functional groups were high, and the active molecules polarity of rhamnolipids molecules were high, so there exists some steric hindrance between rhamnolipids molecules and aromatics molecules, and then the effected was influence.

**Figure 11:**
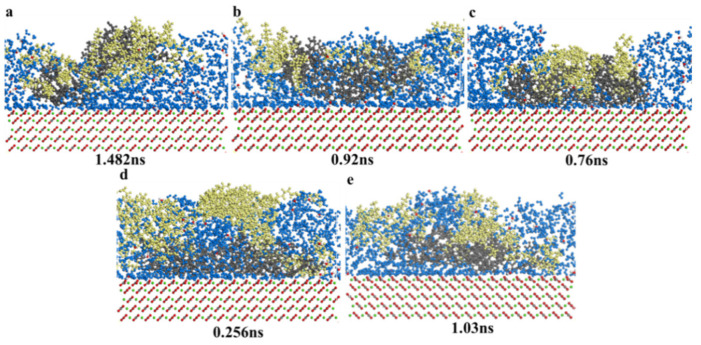
Consecutive snapshot of desorption of aromatics from a modeled calcite surface immersed in (**a**) CTAB; (**b**) SDS; (**c**) TX-100; (**d**) sophorolipid; (**e**) rhamnolipid-water system just right. Blue = water; black = aromatics; red = calcite surface; yellow = surfactants.

Figure 11 showed the consecutive snapshots of desorption of aromatics from a modeled calcite surface immersed in different surfactant solutions, and the total liberation time was labeled. Comparing to saturates, the aromatics liberation was more different than saturates, because the interactive force between aromatics and calcite surface were higher than saturates. The aromatics total liberation time by chemical surfactants (CTAB, SDS, TX-100) and biosurfactants (sophorolipid, rhamnolipid) were as follows: sophorolipid (0.256 ns) < TX-100 (0.76 ns) < SDS (0.92 ns) < rhamnolipid (1.03 ns) < CTAB (1.482 ns). Biosurfactants sophorolipid showed the highest interactive force with aromatics molecules, and chemical surfactants CTAB showed the lowest interactive force with aromatic molecules. Surfactant molecules functional group and structure influenced the surfactant effect with aromatic molecules. 

##### Aromatics Concentration Distribution and MSD 

**Figure 12:**
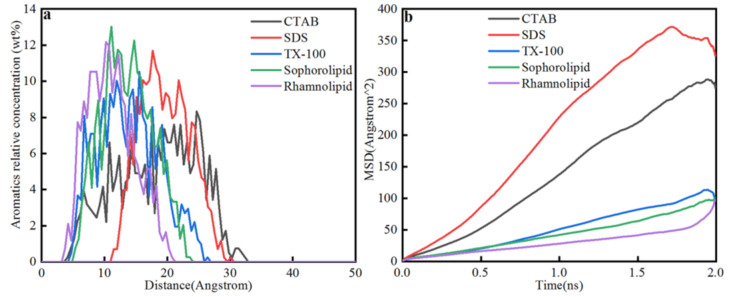
(**a**) Aromatics relative concentration; (**b**) Mean square displacement.

Figure 12 showed the aromatics concentration distribution in five surfactants solutions. When the simulation time reached 2 ns, the system reached the energy balance and temperature balance, and the final aromatics concentration distribution was showed. After 2 ns molecular dynamics simulation, the aromatics were displaced from calcite surface due to the liberation process and diffusion effect, and the displacement order was as follows: SDS (11 Å) > Sophorolipid (5 Å) > TX-100(4.5 Å) > CTAB (4 Å) > Rhamnolipid (3.5 Å). For chemical surfactants, aromatic concentration in CTAB, SDS, and TX-100 solutions were mainly centered at 4.5–32 Å, 11–28.5 Å, and 4.5–26 Å. For the two biosurfactants, aromatics in sophorolipid, rhamnolipid solutions were mainly distributed 5–23 Å and 3.5–21 Å. The aromatics in CTAB solutions displacement was the highest, which means that CTAB makes the aromatics far calcite surface. The average displacement distribution did not accord with the aromatics total liberation time, and the aromatics total liberation time was sophorolipid (0.256 ns) < TX-100 (0.76 ns) < SDS (0.92 ns) < rhamnolipid (1.03 ns) < CTAB (1.482 ns). The total liberation time was the aromatics liberated from calcite surface just right, and the concentrations distribution was the aromatics concentration (2 ns). Aromatics displacement in five surfactants were different due to the different molecules’ diffusion effect. As was shown in Figure 12b, the diffusion coefficient in five surfactants were as follows: SDS > CTAB > TX-100 > sophorolipid > rhamnolipid, and the order accords with the aromatic molecule displacement of the calcite surface. 

##### Aromatics Energy/Temperature Balance

The energy/temperature balance figure was shown in Figure S4. The red line represents kinetic energy, and the kinetic energy remained stable, and non-bond energy, potential energy and total energy decreased with simulation time proceeded. The non-bond energy, potential energy, and total energy decreased significantly within 0.1 ns, and when the simulation time was higher than 0.1 ns, the three energies decreased gentle, and the energy became stable when time reached 1.5 ns. When aromatics dispersed into five surfactants solutions, the temperature stabilized at 298K, and the temperature fluctuation range was within 10 K. Figure S4 indicated that when the simulation time reached 2 ns, aromatics in five surfactants solutions reached energy balance and temperature balance.

#### 3.3.3. Resins–Solid Separation Process

##### Resins Liberation Conformation Analysis

Consecutive snapshot of spontaneous desorption of resins from a modeled calcite surface immersed in five surfactant solutions was shown in Figure S5. Resins have higher molecular weight than saturates and aromatics, and the molecules stacking degree increased. As was shown in Figure S5, similar to the saturates and aromatics, chemical surfactants (CTAB, SDS, TX-100) and biosurfactants (rhamnolipid, sophorolipid) interacted with resins, and they did not interact with calcite surface. Surfactant molecules would be near to the resin molecules surface, and then surfactants would draw the resin molecules far away from the calcite surface under the molecules thermal motion. Resin molecules would be concentrated and aggregated. For CTAB surfactants, when the simulation time was 0.25 ns, only some CTAB surfactant molecules adsorbed onto the resins surface. When the simulation proceeds, more CTAB molecules adsorbed onto resins surface, and resins conformation would be changed. CTAB molecules adsorbed onto resin surfaces from the left side and right side. The other surfactants role was similar to CTAB surfactants. Then the surfactant molecules would adsorb the resin molecules surface from both sides, and the left and right side would liberate from calcite surface preferentially, and the middle part would liberate later. When resins totally liberated from calcite surface, surfactant molecules would continue to interact with resin molecules, and then resin molecules would be far away from calcite surface, and then the resins would diffuse into surfactant solutions. Chemical surfactants (CTAB, SDS, TX-100) molecular weight was small, and the molecular diameter was small, CTAB, SDS, TX-100 would adsorb onto resins surface, and the incomplete adsorption would occur. Surfactant molecules could tow resin molecules to liberate from calcite surface under molecular thermal motion. Biosurfactants sophorolipid have high molecular weight, and the sophorolipid steric hindrance was high, so sophorolipid molecules would adsorb onto resin surfaces, and then surfactants and resins would interact with each other. Rhamnolipids molecules were small, and the adsorption site was dispersed. Therefore, rhamnolipids could help resins liberate from calcite surface, and the displacement of resin molecules would increase.

**Figure 13:**
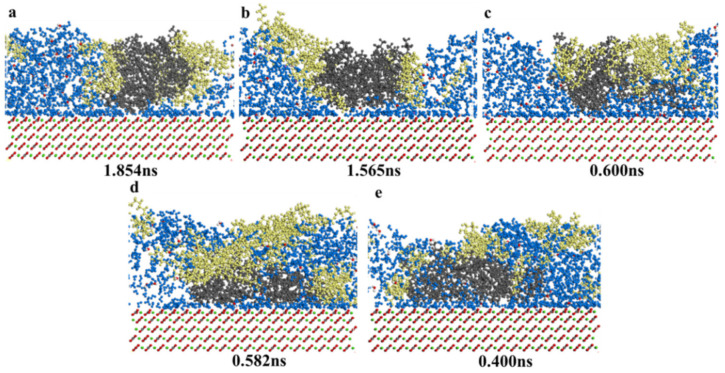
Consecutive snapshot of desorption of resins from a modeled calcite surface immersed in (**a**) CTAB; (**b**) SDS; (**c**) TX-100; (**d**) Sophorolipid; (**e**) Rhamnolipid-water system just right. Blue = water; black = resins; red = calcite surface; yellow = surfactants.

Figure 13 showed the resins’ total liberation from calcite surface in five surfactants solutions. The total liberation time of chemical surfactants (CTAB, SDS, TX-100) and biosurfactants (sophorolipid, rhamnolipid) enhanced resin liberation from calcite surface as follows: rhamnolipid (0.4 ns) <sophorolipid (0.582 ns) < TX-100 (0.600 ns) < SDS (1.565 ns) <CTAB (1.854 ns). The interaction energy between biosurfactants (sophorolipid, rhamnolipid) and resins was high, and the interaction energy between chemical surfactants (CTAB, SDS, TX-100) and resins was low. Rhamnolipid molecules’ functional groups included hydroxyl, ether group, and ester, and these oxygens containing functional groups could interact with resin molecules, which helped resins liberated from calcite surface.

##### Resins Concentration Distribution and MSD 

**Figure 14:**
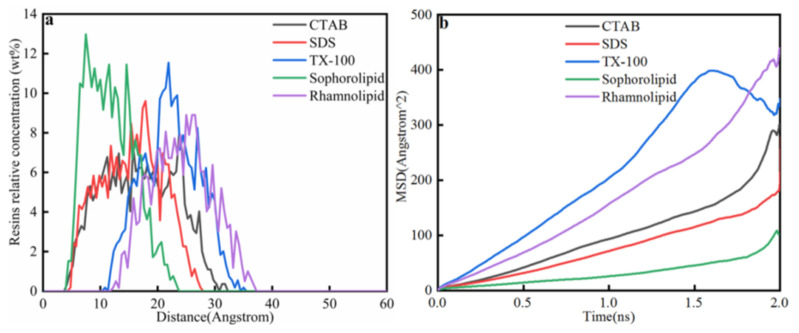
(**a**) Resins relative concentration; (**b**) Mean square displacement.

Figure 14 showed that resins relative concentration in five surfactants solutions. The displacement of resins in five surfactants were different, and the displacement was as follows: rhamnolipid (12.5 Å) > TX-100 (11.5 Å) > SDS (5 Å) > CTAB (4.5 Å) = sophorolipid (4.5 Å). For chemical surfactants, resins in CTAB, SDS, TX-100 solutions mainly concentrated at 4.5–32 Å, 5–27.5 Å, 10.5–35.5 Å. For biosurfactants, resins in sophorolipid, rhamnolipid solutions mainly concentrated at 4.5–22.5 Å and 12.5–37.5 Å. Resins in rhamnolipids showed the highest displacement, which means that rhamnolipid showed the optimal effect on resins. The total liberation time of resins from calcite surface was as follows: rhamnolipid (0.4 ns) <sophorolipid (0.582 ns) < TX-100 (0.600 ns) < SDS (1.565 ns) < CTAB (1.854 ns). As was shown in Figure 14b, the diffusion coefficients of resins in five surfactants solutions were as follows: TX-100 > rhamnolipid > CTAB > SDS > sophorolipid, which indicated that resins diffused into surfactant solutions was different from that resins liberated from mineral surfaces. 

##### Resins Energy/Temperature Balance

The resins in different solution energy balances and temperature balances was shown in Figure S6. The red line represented kinetic energy, which remained stable. When the simulation time was lower than 1.5 ns, non-bond energy, potential energy, and total energy decreased with the simulation time proceeded. However, when the time was higher than 1.5 ns, the non-bond energy, potential energy, and total energy remained stable. When resins were dispersed into five solutions, temperature remained 298 K, and the temperature range was within 10 K. Figure S6 indicated that when the simulation time reached 2 ns, resins in five surfactant solutions attained temperature balance and energy balance. 

#### 3.3.4. Asphaltenes-Solid Separation Process

##### Asphaltene Liberation Conformation Analysis

Asphaltenes showed the highest molecule weight, the highest viscosity, the most complex components and the highest oil–solid interactive force with minerals surface. Literature indicated that asphaltenes were hard to liberate from calcium carbonate surface. In this study, the simulation dynamic time was set 6 ns, which was 3 times the time required for saturates, aromatics, and resins to totally liberate. However, asphaltenes were not totally separate from calcite surface.

Asphaltenes liberated from calcium surface in five surfactant solutions (not total liberation), as was shown in Figure S7. All surfactant movement behaviors were similar, and the surfactant molecules would move to asphaltenes molecules, and then interact with asphaltenes molecules, and then asphaltene molecules would liberate from minerals, but the effect was not so obvious. All surfactants were not directly interacted with calcite surface. Five surfactants adsorbed onto calcite surface, and then tow asphaltenes liberated from calcite surface.

Chemical surfactants (CTAB, SDS, TX-100) would help asphaltenes liberate from calcite surface after 6 ns molecular dynamic simulation. However, there still some asphaltenes would not liberate from calcite surface, and the asphaltenes would not totally liberation. Different from chemical surfactants, biosurfactants (sophorolipid, rhamnolipid) effect on asphaltenes-solid separation was weak. After the molecular dynamic simulation, some asphaltene molecules adsorbed onto calcite surface. Sophorolipid molecules had huge steric hindrance, and sophorolipid could not totally interact with asphaltenes. Rhamnolipid molecules had many oxygen containing functional groups. Therefore, biosurfactants played an unclear role in the oil–solid separation process.

##### Asphaltene Concentration Distribution and MSD 

**Figure 15:**
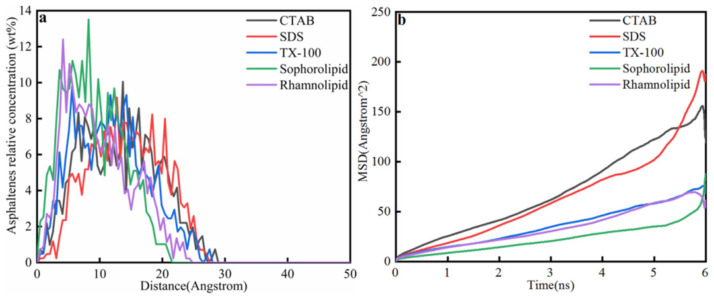
(**a**) Asphaltenes relative concentration; (**b**) Mean square displacement.

Figure 15a showed the asphaltene concentration after 6 ns molecular dynamic simulation. After 6 ns molecular dynamic simulation, the asphaltenes would not totally liberate from calcite surface, and the displacement of asphaltenes in five different surfactants: CTAB (29 Å) > TX-100 (28 Å) ≥ SDS (27.5 Å)>Rhamnolipid (25 Å) > Sophorolipid (21.5 Å). The displacement of asphaltenes in chemical surfactants was higher than that in biosurfactants.

As was shown in Figure 15b, the diffusion coefficient of asphaltenes in five surfactants was as follows: SDS > CTAB > TX-100 > rhamnolipid > sophorolipid. Therefore, surfactants have obvious effect on asphaltenes liberation process, and asphaltenes diffusion role was higher than the surfactants liberation process.

##### Asphaltenes Energy/Temperature Balance

Asphaltenes in different solutions liberation energy balance and temperature balance were shown in Figure S8. The red line represents kinetic energy, and the red line remained stable with time. Non-bond, potential energy, and total energy decreased with the simulation proceeded, and when the simulation time reached 5 ns, the three energies reached stable. Temperatures of asphaltenes in different solutions stabilized at 298 K, and the temperature range was lower than 10 K. Figure S8 indicated that when the simulation time reached 6 ns, asphaltenes in five surfactants solutions reached energy balance and temperature balance. However, asphaltenes were not totally liberated from calcite surface within 6 ns, which is in accord with the literature results. 

#### 3.3.5. Surfactant Functional Group Analysis

Table 8 showed the different surfactants role on heavy oil different components. The optimal effect component for CTAB was saturates, and the total liberation time was 0.420 ns, the second-best component for CTAB was aromatics, and the total liberation time was 1.482 ns. The functional group of CTAB was -N^+^, and -N^+^ could adsorb the saturates surface, the interaction effect of -N^+^ with -S- and benzene ring was weak. The optimal effect component for SDS was aromatics, and the total liberation time was 0.920 ns, the second-best component for SDS was saturates, and the total liberation was 1.180 ns. The functional groups of aromatics were -S- and C_6_H_5_-, the -OSO_3_- functional group in SDS could interact with -S- and C_6_H_5_- (aromatics), because resins contained thiophene -S-, and the interaction between SDS and thiophene -S- was weak. The optimal effect component for TX-100 was resins, and the total liberation time was 0.600 ns, the second-best component was aromatics, and the total liberation was 0.760 ns. The functional groups of TX-100 were -OH and R-O-R’ have strong interaction effect with -S- and C_6_H_5_-. The optimal effect component was aromatics, and the total liberation time was 0.256 ns, the second-best component was resins, and the total liberation time was 0.586 ns. The functional groups of sophorolipid were -OH, R-O-R’, and >C=C<, and the conjugation effect of C_6_H_5_- (aromatics); therefore, aromatics were easy to liberate from calcite surface. The optimal component for rhamnolipid was resins, and the total liberation time was 0.400 ns, the second-best component was aromatics, and the total liberation time was 1.030 ns. The functional groups of rhamnolipid were -OH, -COOH, and R-O-R’, which could interact with -S- (in resins), and therefore, the resins were easy to liberate from calcite surface. The optimal surfactant for saturates was CTAB, the optimal surfactant for aromatics was rhamnolipid, and the optimal surfactant for resins was rhamnolipid. -N^+^ has strong interact role with saturates, -OH, -COOH, R-O-R’ have strong specific effect with -S-, and >C=C< has specific effect with benzene ring. Different surfactants have different action effects for bitumen SARA components, and therefore, the research results provide a theoretical basis for surfactant formulation.

According to the results of five surfactants enhanced heavy oil–solid separation, the SARA content of five different bitumen was as follows. The saturates content was as follows: CTAB (17.21 wt %) > sophorolipid (17.14 wt %) > TX-100 (16.89 wt %) > SDS (16.58 wt %) > rhamnolipid (16.34 wt %). The aromatics content was as follows: sophorolipid (30.04 wt %) > TX-100 (29.86 wt %) > SDS (29.71 wt %)>rhamnolipid (29.56 wt %) > CTAB (29.12 wt %). The resins content was as follows: rhamnolipid (30.12 wt %) > sophorolipid (29.37 wt %) > SDS (29.33 wt %) > TX-100 (29.25 wt %) > CTAB (29.19 wt %). The asphaltenes content was as follows: CTAB (24.48 wt %) > SDS (24.38 wt %) > TX-100 (24.00 wt %) > rhamnolipid (23.98 wt %) > sophorolipid (23.45 wt %).

For saturates and aromatics, the molecular dynamic simulation results coincide with the experiment, which indicated that the simulation results were acceptable. Namely, the lower time for bitumen component liberation from a mineral’s surface, the higher the SARA content. However, for asphaltenes and resins, there exist some errors between experiment results and simulation results.

### 3.4. Surfactant-Enhanced Oil Recovery Mechanism

Surfactants could effectively increase heavy oil recovery and B/S ratio. The reasons were as follows. Firstly, surfactants would effectively solubilize heavy oil components [48,49]. Then, surfactants could decrease oil–solid interaction force (Figure 5, Figure 6, Figure 7 and Figure 8, Table 4, Table 5, Table 6 and Table 7). Last but not least, molecular dynamic simulation showed that five surfactants could effectively adsorb onto heavy oil component surfaces, and then surfactants would drag the heavy oil components far away from the calcite surface (Figure 9, Figure 11 and Figure 13). When surfactants were used to enhance reactive extraction, the surfactants would help the acid solution pour into oil–solid interface, and then the carbonate asphalt rocks dissolved into acid solution would be enhanced. Then the HCl solution would effectively react with CaCO_3_.

## 4. Conclusions

In this study, we used surfactants to enhance HCl reaction extraction. The surfactant role mechanism was studied by SARA analysis, element analysis, oil–solid interaction force measurement, and molecular dynamics simulation. The detailed conclusions were as follows.

(1) CTAB, SDS, TX-100, sophorolipid, and rhamnolipid were used to enhance the reactive extraction process, which increased bitumen recovery and B/S.
(2) When surfactants were used to enhance-HCl reaction extraction, the heavy components content would increase, and light components content would decrease. Surfactants could decrease oil–solid interaction force. The oil–solid interaction force changes with surfactant species, concentration, pH, asphaltenes content, and salinity.
(3) The liberation process of chemical surfactants (CTAB, SDS, TX-100) and biosurfactant (sophorolipid, rhamnolipid) effect on saturates, aromatics, resins, and asphaltenes were similar. The surfactant molecules would adsorb onto the SARA components surface, and then the surfactant molecules would tow the asphaltenes liberated from calcite surface, all the surfactants would not adsorb onto the calcite surface.
(4) Saturates, aromatics and resins could reach the energy balance and temperature balance within 2 ns, and the three components could totally liberate from calcite surface. However, the simulation time for asphaltenes was 6 ns, asphaltenes cannot totally liberate from calcite surface, and the results were in accordance with the published literature. When saturates, aromatics and resins three components liberated from calcite surface in five surfactants solutions, the diffusion coefficients were similar to the displacement.
(5) Saturates, aromatics, resins, and asphaltenes in five surfactants solutions liberation effect varied significantly. The saturates in five surfactant solutions’ liberation effect was: CTAB > sophorolipid> TX-100 > SDS > rhamnolipid, the aromatics in five surfactant solutions’ effect was: sophorolipid> TX-100 > SDS> rhamnolipid > CTAB, the resins in five surfactant solutions’ liberation ability was: rhamnolipid > sophorolipid > TX-100 > SDS > CTAB, and the asphaltenes in five surfactant solutions’ effect was: CTAB > SDS > TX-100 > sophorolipid > rhamnolipid.
(6) The molecular dynamic simulation results were similar to the experiment results, which identified the simulation effectiveness, and provided the theory for future surfactant combination for oil–solid separation.

## Figures and Tables

**Table 1:** Element analysis of SiO_2_ sphere covered with heavy oil.

Element	Percentage by Weight	Percentage by Atom
C	54.33	63.96
O	34.30	30.31
Si	11.37	5.73

**Table 2:** SARA analysis of heavy oil by different surfactants enhanced reaction extraction process.

Surfactants	SARA Content of the Bitumen from Different Surfactants-Enhanced Process			
	Saturates (wt %)	Aromatics (wt %)	Resins (wt %)	Asphaltenes (wt %)
CTAB	17.21	29.12	29.19	24.48
SDS	16.58	29.71	29.33	24.38
TX-100	16.89	29.86	29.25	24.00
Sophorolipid	17.14	30.04	29.37	23.45
Rhamnolipid	16.34	29.56	30.12	23.98
HCl	17.78	20.69	28.89	22.64
Soxhlet extraction	18.07	31.28	28.32	22.33

**Table 3:** Element analysis of seven different bitumen samples.

Sample	C (wt %)	H (wt %)	Other Elements (wt %)	C/H	Surfactants
Bitumen ^a^	80.795	9.527	9.678	8.48	Soxhlet extraction
Bitumen ^b^	81.242	9.423	9.335	8.62	HCl
Bitumen ^c^	82.408	9.027	8.565	9.13	CTAB
Bitumen ^d^	81.634	9.256	9.110	8.82	SDS
Bitumen ^e^	81.947	9.164	8.889	8.94	TX-100
Bitumen ^f^	82.245	9.114	8.641	9.02	Sophorolipid
Bitumen ^g^	81.873	9.218	8.909	8.88	Rhamnolipid

Note: Bitumen from Soxhelt extraction (^a^), HCl reaction extraction (^b^), CTAB-enhanced (^c^), SDS-enhanced (^d^), TX-100-enhanced (^e^), Sophorolipid-enhanced (^f^), and Rhamonilipid-enhanced (^g^)-HCl reaction extraction.

**Table 4:** Adhesion forces (F/R) between heavy oil and calcium carbonate in different surfactants concentrations at ambient conditions (25 °C, C_KCl_ = 1mmol/L, pH = 3.5, 45 ppm asphaltenes; F/R, mN/m).

Surfactant Types	Surfactant Concentration (mmol/L)				
	0.001	0.01	0.05	0.1	0.5
CTAB	0.72	0.42	0.13	—	—
SDS	1.41	1.36	0.74	0.47	—
TX-100	2.64	0.89	0.85	0.63	0.10
Sophorolipid	0.91	0.39	0.36	0.10	—
Rhamnolipid	1.52	1.02	0.56	0.48	—

**Table 5:** Adhesion forces (F/R) between heavy oil and calcium carbonate in different pH at ambient conditions (0.01 mmol/L surfactants, 25 °C, 1 mmol/L KCl, 45 ppm asphaltenes; F/R, mN/m).

Surfactant Species	pH			
	3.5	5.5	8.5	10.5
CTAB	0.42	0.01	—	—
SDS	1.36	0.57	0.13	—
TX-100	0.89	0.67	0.60	0.57
Sophorolipid	0.39	0.12	—	—
Rhamnolipid	1.02	0.36	0.21	0.10

**Table 6:** Adhesion forces (F/R) between heavy oil and calcium carbonate in different asphaltenes concentration surfactants at ambient conditions (0.01 mmol/L surfactants, 25 °C, 1 mmol/L KCl, pH = 3.5; F/R, mN/m).

Surfactant Species	Asphaltenes Content (ppm)			
	45	70	95	145
CTAB	0.42	1.11	1.71	2.56
SDS	1.36	2.18	2.23	3.39
TX-100	0.89	1.34	2.00	2.84
Sophorolipid	0.39	0.59	0.87	1.80
Rhamnolipid	1.02	1.07	1.36	2.21

**Table 7:** Adhesion forces (F/R, mN/m) between heavy oil and calcium carbonate in different salinities at ambient conditions (0.01 mmol/L surfactants, 25 °C, pH = 3.5, 45 ppm asphaltenes; F/R, mN/m).

Surfactants Species	Salinities				
	C_KCl_ = 1 mmol/L	C_KCl_ = 2 mmol/L	C_KCl_ = 5 mmol/L	C_CaCl2_ = 2 mmol/L	C_CaCl2_ = 5 mmol/L
CTAB	—	0.01	0.04	0.64	1.03
SDS	—	0.16	0.22	0.61	1.05
TX-100	—	0.32	0.70	1.31	2.10
Sophorolipid	—	0.18	0.36	0.40	0.58
Rhamnolipid	—	0.02	0.26	0.46	0.76

**Table 8:** Different surfactants’ effects on heavy oil components liberation time.

Surfactants Species	Functional Group	Carbon Number	Heavy Oil Component	Liberation Time (ns)	Functional Group	Benzene Ring	Molecular Weight	Effect
CTAB	-N^+^	19	Saturates	0.420	-	-	282	The optimal component
SDS	-OSO_3_^-^	12	Aromatics	0.920	-S-, C_6_H_5_-	7	634	
TX-100	-OH, R-O-R’ (2)	18	Resins	0.600	-S-, C_6_H_5_-	2	712	
Sophorolipid	-OH (5), -COOH, R-O-R’ (6),>C=C<	32	Aromatics	0.256	-S-, C_6_H_5_-	7	634	
Rhamnolipid	-OH (3), -COOH, R-O-R’ (2)	16	Resins	0.400	-S-, C_6_H_5_-	2	712	
CTAB	-N^+^	19	Aromatics	1.482	-S-, C_6_H_5_-	7	634	The second-best component
SDS	-OSO_3_-	12	Saturates	1.180	-	-	282	
TX-100	-OH, R-O-R’ (2)	18	Aromatics	0.760	-S-, C_6_H_5_-	7	634	
Sophorolipid	-OH (5), -COOH, R-O-R’ (6),>C=C<	32	Resins	0.582	-S-, C_6_H_5_-	2	712	
Rhamnolipid	-OH (3), -COOH, R-O-R’ (2)	16	Aromatics	1.030	-S-, C_6_H_5_-	7	634	
CTAB	-N^+^	19	Resins	1.854	-S-, C_6_H_5_-	2	712	The last component
SDS	-OSO_3_^-^	12	Resins	1.565	-S-, C_6_H_5_-	2	712	
TX-100	-OH, R-O-R’ (2)	18	Saturates	0.816	-	-	282	
Sophorolipid	-OH (5), -COOH, R-O-R’ (6),>C=C<	32	Saturates	0.662	-	-	282	
Rhamnolipid	-OH (3), -COOH, R-O-R’ (2)	16	Saturates	1.520	-	-	282	

## Data Availability

The data is included in the main text and the supplementary materials.

## Supplementary Materials

The following are available online at https://www.mdpi.com/article/10.3390/nano11071835/s1, Figure S1: Consecutive snapshot of spontaneous desorption of saturates from a modelled calcite surface immersed in (a) CTAB; (b) SDS; (c) TX-100; (d) Sophorolipid; (e) Rhamnolipid-water system, Figure S2: The change of saturates surfactants solutions system energy and temperature with time (a,b) CTAB; (c,d) SDS; (e,f) TX-100; (g,h) sophorolipid; (i,j) Rhamnolipid, Figure S3: Consecutive snapshot of spontaneous desorption of aromatics from a modelled calcite surface immersed in (a) CTAB; (b) SDS; (c) TX-100; (d) Sophorolipid; (e) Rhamnolipid-water system, Figure S4: The change of aromatics surfactants solutions system energy and temperature with time (a,b) CTAB; (c,d) SDS; (e,f) TX-100; (g,h) sophorolipid; (i,j) Rhamnolipid, Figure S5: Consecutive snapshot of spontaneous desorption of resins from a modelled calcite surface immersed in (a) CTAB; (b) SDS; (c) TX-100; (d) Sophorolipid; (e) Rhamnolipid-water system, Figure S6: The change of resins system energy and temperature with time (a,b) CTAB; (c,d) SDS; (e,f) TX-100; (g,h) sophorolipid; (i,j) Rhamnolipid, Figure S7: Consecutive snapshot of spontaneous desorption of asphaltenes from a modelled calcite surface immersed in (a) CTAB; (b) SDS; (c) TX-100; (d) Sophorolipid; (e) Rhamnolipid-water system, Figure S8: The change of asphaltenes system energy and temperature with time (a,b) CTAB; (c,d) SDS; (e,f) TX-100; (g,h) sophorolipid; (i,j) Rhamnolipid.

## Abbreviations

saturates, aromatics, resins, asphaltenes—SARA; enhanced oil recovery—EOR; miscible enhanced oil recovery—M-EOR; thermal enhanced oil recovery—T-EOR; chemical enhanced oil recovery—C-EOR; hot water based extraction—HWBE; B/S—bitumen/solid mass ratio; cetyltrimethylammonium bromide—CTAB; sodium dodecyl sulfate—SDS; Triton X-100—TX-100; atomic force microscopy—AFM; Materials Studio—MS.

## References

[1] Peng B.L., Zhang L.C., Luo J.H., Wang P.M., Ding B., Zeng M.X., Cheng Z.D. (2017). A review of nanomaterials for nanofluid enhanced oil recovery. RSC Adv..

[2] Zhang N., Yin M.F., Wei M.Z., Bai B.J. (2019). Identification of CO_2_ sequestration opportunities: CO_2_ miscible flooding guidelines. Fuel.

[3] Han J., Han S., Sung W., Lee Y. (2018). Effects of CO_2_ miscible flooding on oil recovery and the alteration of rock properties in a carbonate reservoir. J. CO2 Util..

[4] Ramesh V.K., Chintala V., Kumar S. (2019). Recent developments, challenges and opportunities for harnessing solar renewable energy for thermal Enhanced Oil Recovery (EOR). Energy Sources Part A Recovery Util. Environ. Eff..

[5] Menad N.A., Noureddine Z., Hemmati-Sarapardeh A., Shamshirband S. (2019). Modeling temperature-based oil-water relative permeability by integrating advanced intelligent models with grey wolf optimization: Application to thermal enhanced oil recovery processes. Fuel.

[6] Brantson E.T., Ju B.S., Appau P.O., Akwensi P.H., Peprah G.A., Liu N.N., Aphu E.S., Boah E.A., Borsah A.A. (2020). Development of hybrid low salinity water polymer flooding numerical reservoir simulator and smart proxy model for chemical enhanced oil recovery (CEOR). J. Pet. Sci. Eng..

[7] Gbadamosi A.O., Junin R., Manan M.A., Agi A., Yusuff A.S. (2019). An overview of chemical enhanced oil recovery: Recent advances and prospects. Int. Nano Lett..

[8] Haghighi O.M., Firozjaii A.M. (2020). An experimental investigation into enhancing oil recovery using combination of new green surfactant with smart water in oil-wet carbonate reservoir. J. Pet. Explor. Prod. Technol..

[9] Lu Y., Li R., Manica R., Liu Q., Xu Z.J.A.J. (2021). Enhancing oil–solid and oil–water separation in heavy oil recovery by CO_2_-responsive surfactants. AIChE J..

[10] Zampieri M.F., Ferreira V.H.S., Quispe C.C., Sanches K.K.M., Moreno R. (2020). History matching of experimental polymer flooding for enhanced viscous oil recovery. J. Braz. Soc. Mech. Sci. Eng..

[11] Tanino Y., Syed A. (2019). Enhanced oil recovery by polymer flooding: Direct, low-cost visualization in a Hele-Shaw cell. Educ. Sci..

[12] Fakher S., Abdelaal H., Elgahawy Y., Imqam A. (2019). A characterization of different alkali chemical agents for alkaline flooding enhanced oil recovery operations: An experimental investigation. SN Appl. Sci..

[13] Zhang H.Y., Chen G.Y., Dong M.Z., Zhao S.Q., Liang Z.W. (2016). Evaluation of different factors on enhanced oil recovery of heavy oil using different alkali solutions. Energy Fuels.

[14] Xiang B., Li R., Liu B., Manica R., Liu Q. (2020). Effect of sodium citrate and calcium ions on the spontaneous displacement of heavy oil from quartz surfaces. J. Phys. Chem. C.

[15] Bai T., Grundy J.S., Manica R., Li M., Liu Q. (2020). Controlling the interaction forces between an air bubble and oil with divalent cations and sodium citrate. J. Phys. Chem. C.

[16] Wei P., Pu W.F., Sun L., Pu Y., Li D.B., Chen Y. (2018). Role of water-soluble polymer on foam-injection process for enhancing oil recovery. J. Ind. Eng. Chem..

[17] Wei P., Pu W.F., Sun L., Wang B. (2017). Research on nitrogen foam for enhancing oil recovery in harsh reservoirs. J. Pet. Sci. Eng..

[18] Sagala F., Hethnawi A., Nassar N.N. (2020). Hydroxyl-functionalized silicate-based nanofluids for enhanced oil recovery. Fuel.

[19] Joonaki E., Ghanaatian S. (2014). The application of nanofluids for enhanced oil recovery: Effects on interfacial tension and coreflooding Process. Pet. Sci. Technol..

[20] Cheraghian G., Rostami S., Afrand M. (2020). Nanotechnology in enhanced oil recovery. Processes.

[21] Gbadamosi A.O., Junin R., Manan M.A., Agi A., Oseh J.O., Usman J. (2019). Synergistic application of aluminium oxide nanoparticles and oilfield polyacrylamide for enhanced oil recovery. J. Pet. Sci. Eng..

[22] Cheraghian G. Improved heavy oil recovery by nanofluid surfactant flooding—An experimental study. Proceedings of the 78th EAGE Conference and Exhibition.

[23] Nowrouzi I., Manshad A.K., Mohammadi A.H. (2019). Effects of concentration and size of TiO2 nano-particles on the performance of smart water in wettability alteration and oil production under spontaneous imbibition. J. Pet. Sci. Eng..

[24] Li S.D., Torsaeter O., Lau H.C., Hadia N.J., Stubbs L.P. (2019). The impact of nanoparticle adsorption on transport and wettability alteration in water-wet berea sandstone: An experimental study. Front. Phys..

[25] Amraei A., Fakhroueian Z., Bahramian A. (2014). Influence of new SiO2 nanofluids on surface wettability and interfacial tension behaviour between oil—water Interface in EOR processes. J. Nano Res..

[26] Moghadam T.F., Azizian S. (2014). Synergistic effect of ZnO nanoparticles and triblock copolymer surfactant on the dynamic and equilibrium oil-water interfacial tension. Soft Matter.

[27] Gbadamosi A.O., Junin R., Manan M.A., Agi A., Oseh J.O., Usman J. (2019). Effect of aluminium oxide nanoparticles on oilfield polyacrylamide: Rheology, interfacial tension, wettability and oil displacement studies. J. Mol. Liq..

[28] Liu K.L., Kondiparty K., Nikolov A.D., Wasan D. (2012). Dynamic spreading of nanofluids on solids part II: Modeling. Langmuir.

[29] Wasan D.T., Nikolov A.D. (2003). Spreading of nanofluids on solids. Nature.

[30] Liu Y.F., Qiu Z.S., Zhao C., Nie Z., Zhong H.Y., Zhao X., Liu S.J., Xing X.J. (2020). Characterization of bitumen and a novel multiple synergistic method for reducing bitumen viscosity with nanoparticles and surfactants. RSC Adv..

[31] Zhu Y.L., Lu Y., Liu Q.X., Masliyah J., Xu Z.H. (2020). Comprehensive study on cleaner production of heavy oil from Athabasca oil sands using chemical additives in biodiesel-assisted ambient-aqueous bitumen extraction process. J. Clean. Prod..

[32] Mikula R.J., Munoz V.A., Omotoso O. (2007). Laboratory and pilot experience in the development of a conventional water-based extraction process for the Utah Asphalt Ridge tar sands. J. Can. Pet. Technol..

[33] Lin F., Stoyanov S.R., Xu Y. (2017). Recent advances in nonaqueous extraction of bitumen from mineable oil sands: A review. Org. Process Res. Dev..

[34] Zhang H.Y., Tan X.L., Liu Q. (2021). Fine solids removal from non-aqueous extraction bitumen: A literature review. Fuel.

[35] Joshi V.A., Kundu D. (2021). Ionic liquid promoted extraction of bitumen from oil sand: A review. J. Pet. Sci. Eng..

[36] Hon J.J., Du J.Z., Su H., Sun L.Y. (2020). Biosurfactants assisted enhanced bitumen recovery from carbonate asphalt rocks by using acid washed process. Fresenius Environ. Bull..

[37] Li X.G., Bian R.Z., Wang J.Y., Wang X.Y., Ma J., Ma G.Q., Sui H., He L. (2019). Recovery of extra-heavy oil and minerals from carbonate asphalt rocks by reactive extraction. RSC Adv..

[38] Zhou Y.X., Wu X., Zhong X., Sun W., Pu H., Zhao J.X. (2019). Surfactant-augmented functional silica nanoparticle based nanofluid for enhanced oil recovery at high temperature and salinity. ACS Appl. Mater. Interfaces.

[39] Huang P., Jia H., Han Y., Wang Q., Wei X., Luo Q., Dai J., Song J., Yan H., Liu D. (2020). Designing novel high-performance shale inhibitors by optimizing the spacer length of imidazolium-based bola-form ionic liquids. Energy Fuels.

[40] Lemahieu G., Ontiveros J.F., Souza N.T.T., Molinier V., Aubry J.-M. (2021). Fast and accurate selection of surfactants for enhanced oil recovery by dynamic Salinity-Phase-Inversion (SPI). Fuel.

[41] He Y.F., Liao K.L., Bai J.M., Fu L.P., Ma Q.L., Zhang X., Ren Z.K., Wang W.Y. (2021). Study on a nonionic surfactant/nanoparticle composite flooding system for enhanced oil recovery. ACS Omega.

[42] Zhan F.X., Gong L.Y., Luan H.X., Chen Q.S., Liao G.Z., Feng Y.J. (2021). Enhancing oil recovery by low concentration of alkylaryl sulfonate surfactant without ultralow interfacial tension. J. Surfactants Deterg..

[43] Machale J., Al-Bayati D., Almobarak M., Ghasemi M., Saeedi A., Sen T.K., Majumder S.K., Ghosh P. (2021). Interfacial, emulsifying, and rheological properties of an additive of a natural surfactant and polymer and its performance assessment for application in enhanced oil recovery. Energy Fuels.

[44] Yuan S.D., Wang S.Y., Wang X.Y., Guo M.M., Wang Y.D., Wang D.S. (2016). Molecular dynamics simulation of oil detachment from calcite surface in aqueous surfactant solution. Comput. Theor. Chem..

[45] Li X.F., Xue Q.Z., Wu T.T., Jin Y.K., Ling C.C., Lu S.F. (2015). Oil detachment from silica surface modified by carboxy groups in aqueous cetyltriethylammonium bromide solution. Appl. Surf. Sci..

[46] Li X.F., Xue Q.Z., Zhu L., Jin Y.K., Wu T.T., Guo Q.K., Zheng H.X., Lu S.F. (2016). How to select an optimal surfactant molecule to speed up the oil-detachment from solid surface: A computational simulation. Chem. Eng. Sci..

[47] Liu Y.F., Qiu Z.S., Zhong H.Y., Nie Z., Li J., Huang W.A., Zhao X. (2019). Bitumen recovery from crude bitumen samples from Halfaya Oilfield by single and composite solvents-process, parameters, and mechanism. Materials.

[48] Panumonwatee G., Charoensaeng A., Arpornpong N. (2021). Application of hydrophilic-lipophilic deviation equations to the formulation of a mixed-surfactant washing agent for crude rice bran oil removal from spent bleaching earth. J. Surfactants Deterg..

[49] Noor S., Taj M.B., Ashar A. (2021). Solubilization of cationic dye in single and mixed micellar media. J. Mol. Liq..

