# Supplementary material for: Surfactants Enhanced Heavy Oil–Solid Separation from Carbonate Asphalt Rocks-Experiment and Molecular Dynamic Simulation
Source: Nanomaterials (Basel). 2021 Jul 14;11(7):1835. doi: 10.3390/nano11071835 (PMC8308391; doi:10.3390/nano11071835)
Supplement: Supplementary file 1 [file nanomaterials-11-01835-s001.zip › nanomaterials-1288208-supplementary.pdf]

nanomaterials

MDPI

Article

Surfactants Enhanced Heavy Oil–Solid Separation from Carbonate Asphalt Rocks-Experiment and Molecular Dynamic Simulation

Jinjian Hou [1,2,3], Jinze Du [1,2,*], Hong Sui [1,2,3,*] and Lingyu Sun [1,2,3]

1 School of Chemical Engineering and Technology, Tianjin University, Tianjin 300072, China; houjinjian@tju.edu.cn (J.H.); sunlingyu4321@163.com (L.S.)
2 National Engineering Research Centre of Distillation Technology, Tianjin 300072, China
3 Collaborative Innovation Center of Chemical Science and Engineering, Tianjin 300072, China
* Correspondence: tjdujinze@hotmail.com (J.D.); suihong@tju.edu.cn (H.S.)

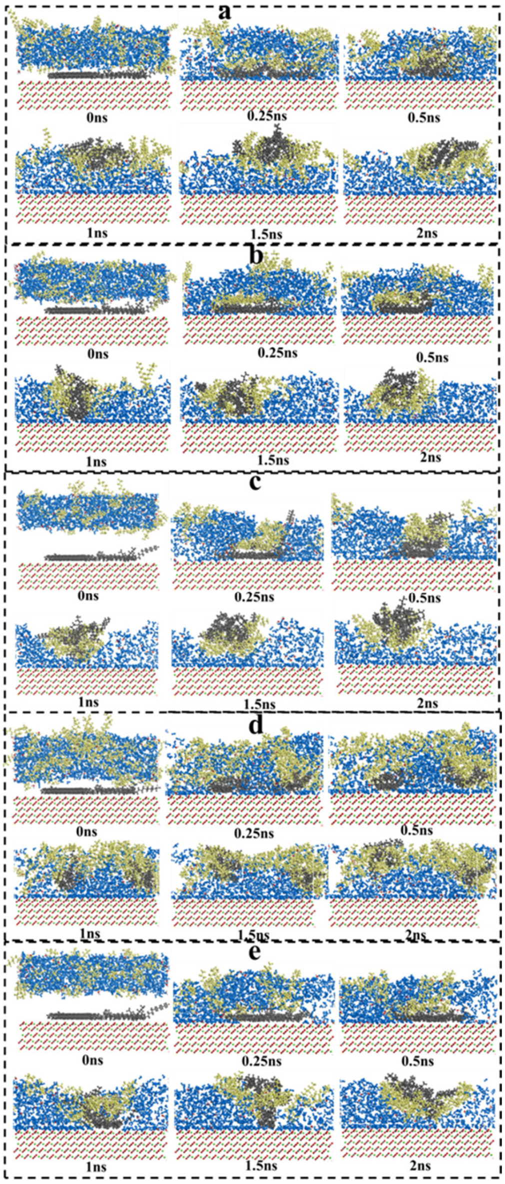

Figure S1. Consecutive snapshot of spontaneous desorption of saturates from a modelled calcite surface immersed in (**a**) CTAB; (**b**) SDS; (**c**) TX−100; (**d**) Sophorolipid; (**e**) Rhamnolipid-water system. Blue = water; black = saturates; red and green = calcite surface; yellow = surfactants.

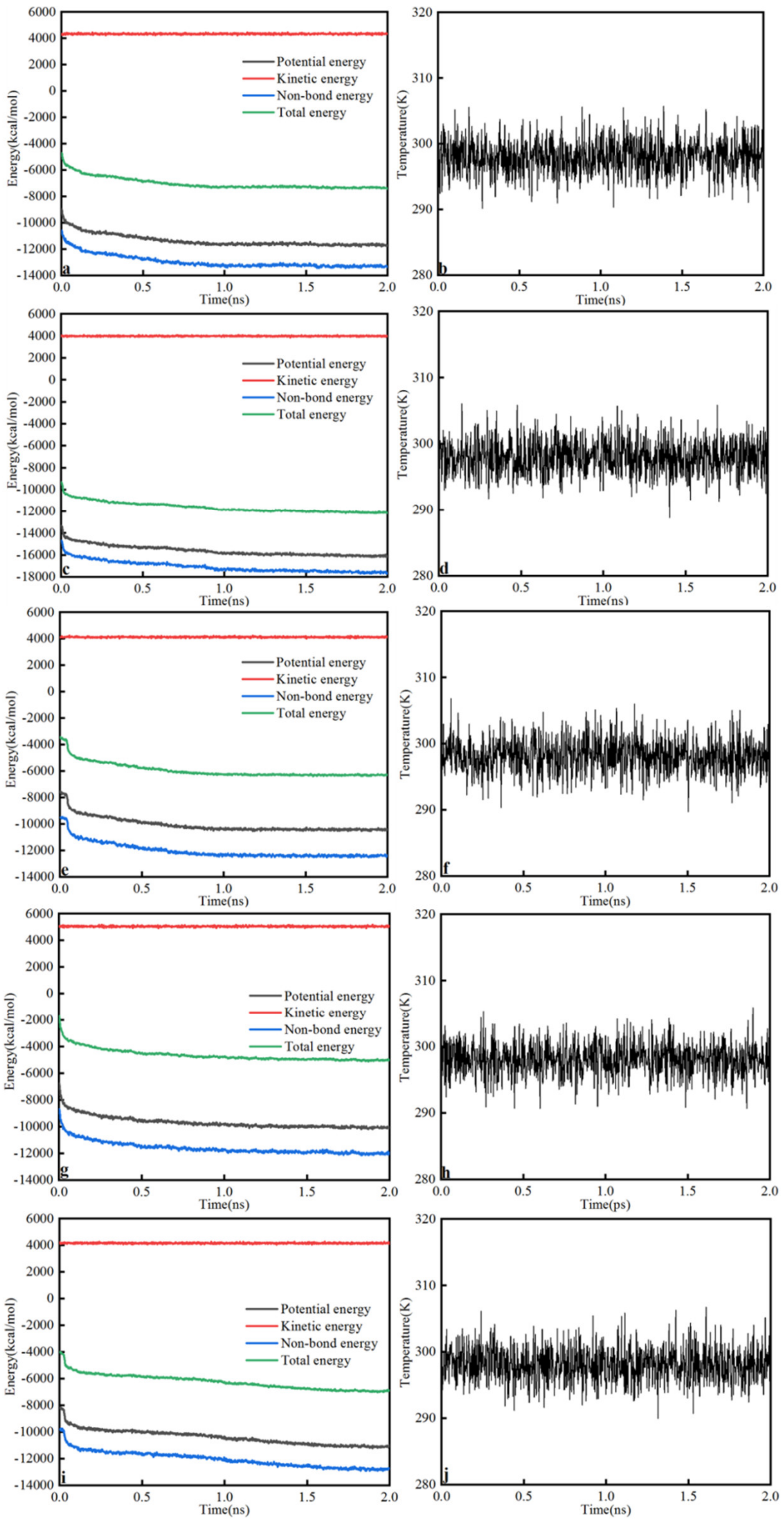

Figure S2. The change of saturates surfactants solutions system energy and temperature with time (**a**,**b**) CTAB; (**c**,**d**) SDS; (**e**,**f**) TX−100; (**g**,**h**) sophorolipid**;** (**i**,**j**) Rhamnolipid.

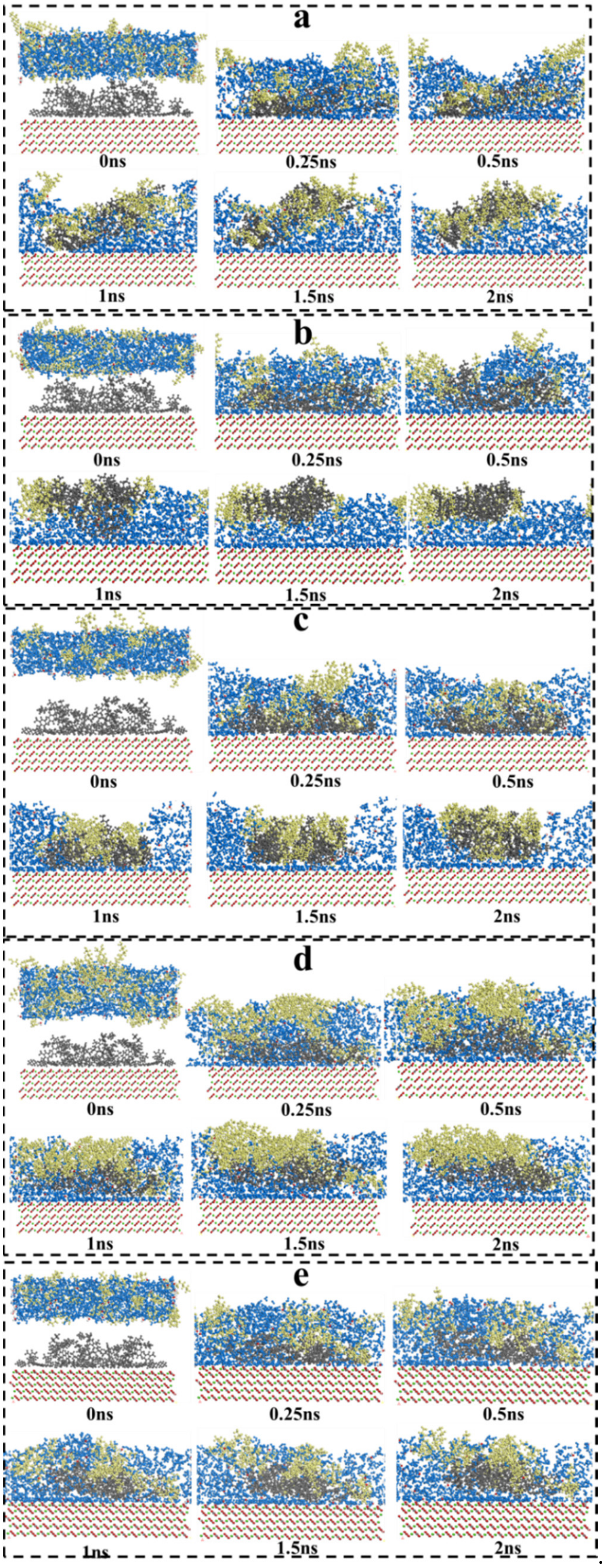

Figure S3. Consecutive snapshot of spontaneous desorption of aromatics from a modelled calcite surface immersed in (**a**) CTAB; (**b**) SDS; (**c**) TX−100; (**d**) Sophorolipid; (**e**) Rhamnolipid-water system. Blue = water; black = aromatics; red and green = calcite surface; yellow = surfactants.

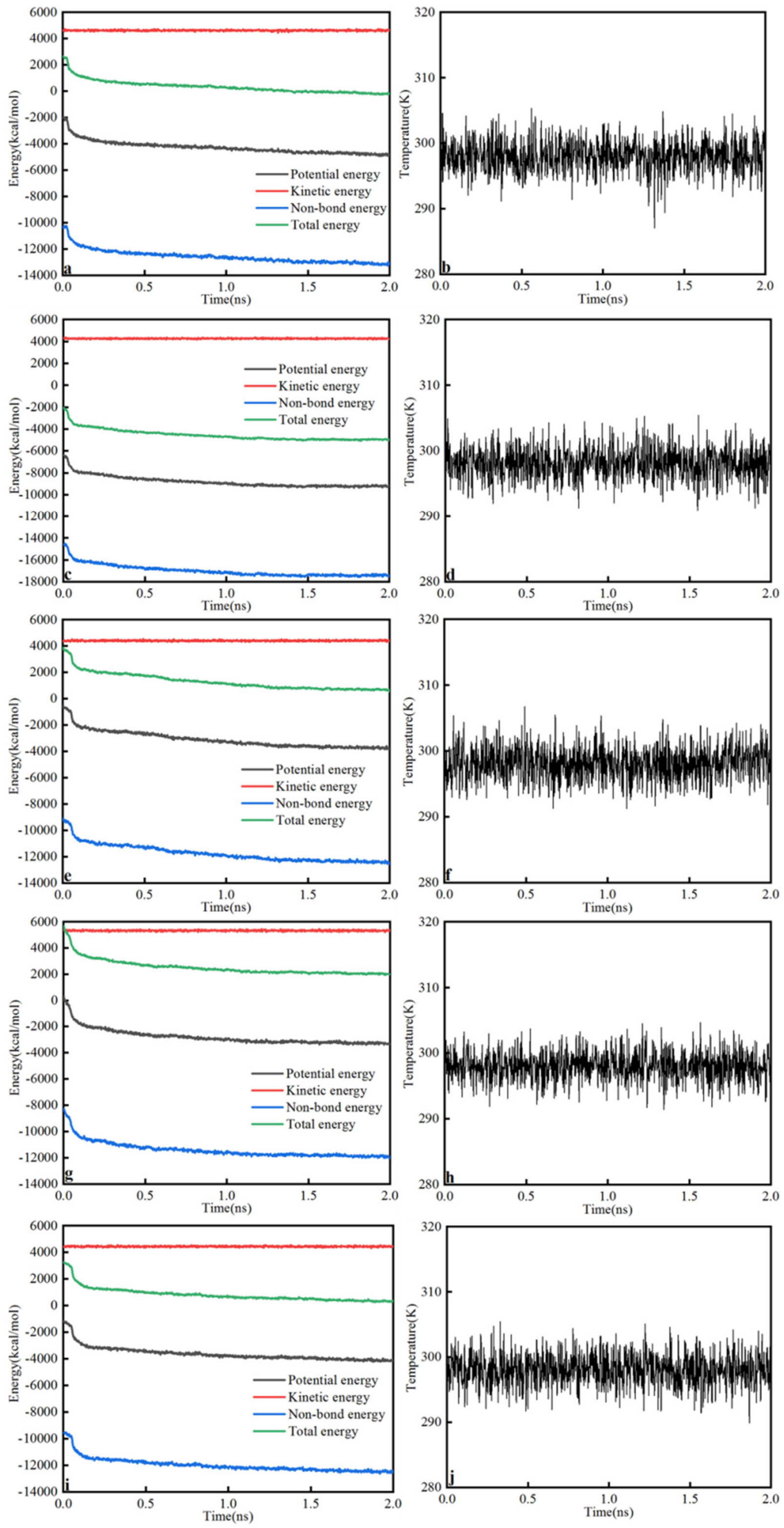

Figure S4. The change of aromatics surfactants solutions system energy and temperature with time (**a**,**b**) CTAB; (**c**,**d**) SDS; (**e**,**f**) TX−100; (**g**,**h**) sophorolipid; (**i**,**j**) Rhamnolipid.

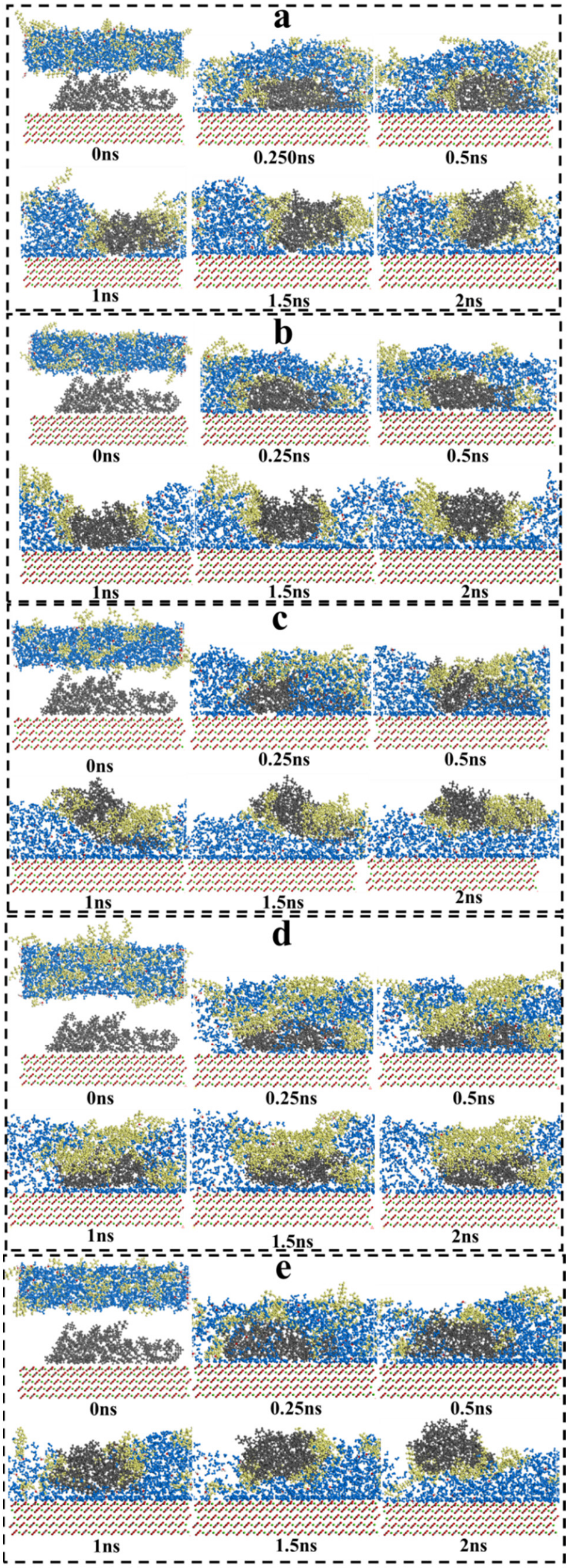

Figure S5. Consecutive snapshot of spontaneous desorption of resins from a modelled calcite surface immersed in (**a**) CTAB; (**b**) SDS; (**c**) TX−100; (**d**) Sophorolipid; (**e**) Rhamnolipid-water system. Blue = water; black = resins; red and green = calcite surface; yellow = surfactants.

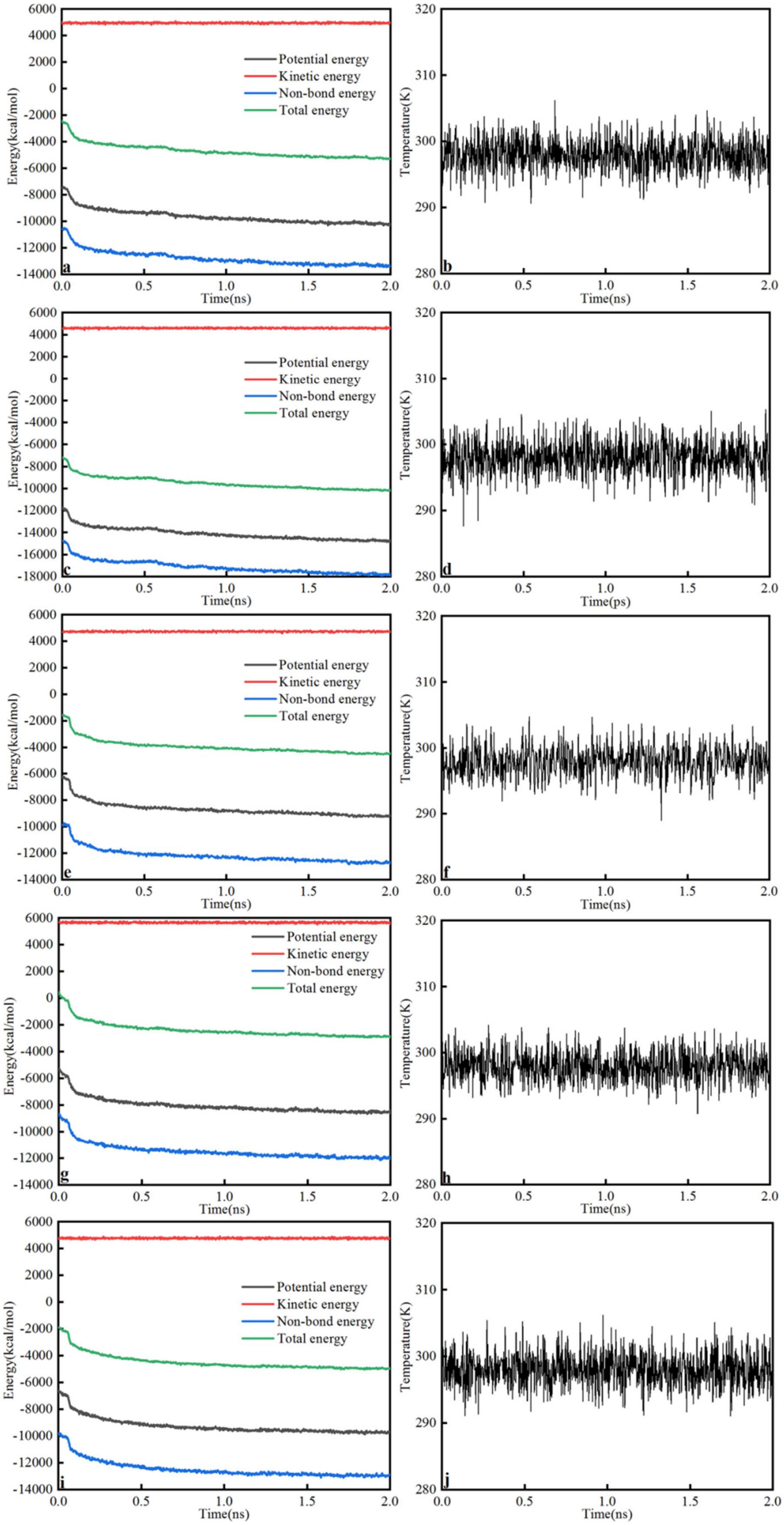

Figure S6. The change of resins system energy and temperature with time (**a**,**b**) CTAB; (**c**,**d**) SDS; (**e**,**f**) TX−100; (**g**,**h**) sophorolipid; (**i**,**j**) Rhamnolipid.

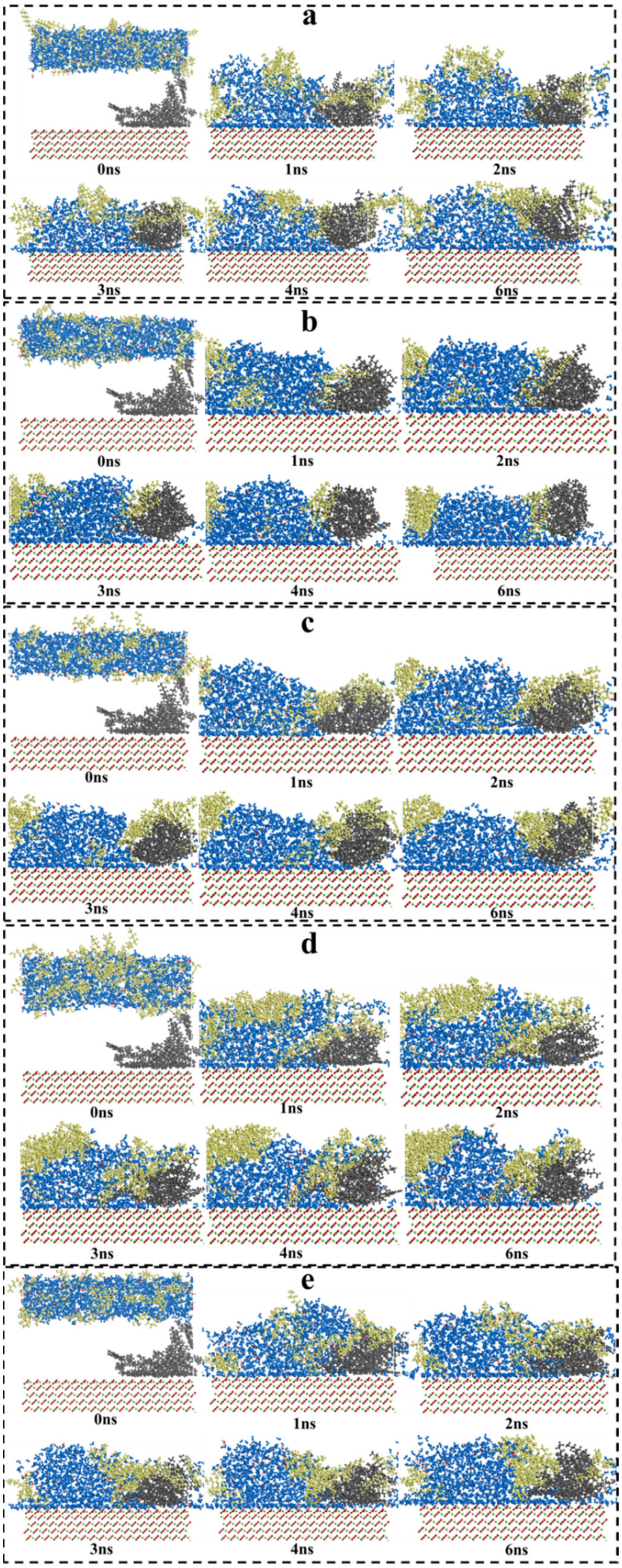

Figure S7. Consecutive snapshot of spontaneous desorption of asphaltenes from a modelled calcite surface immersed in (**a**) CTAB; (**b**) SDS; (**c**) TX−100; (**d**) Sophorolipid; (**e**) Rhamnolipid-water system. Blue = water; black = asphaltenes; red and green = calcite surface; yellow = surfactants.

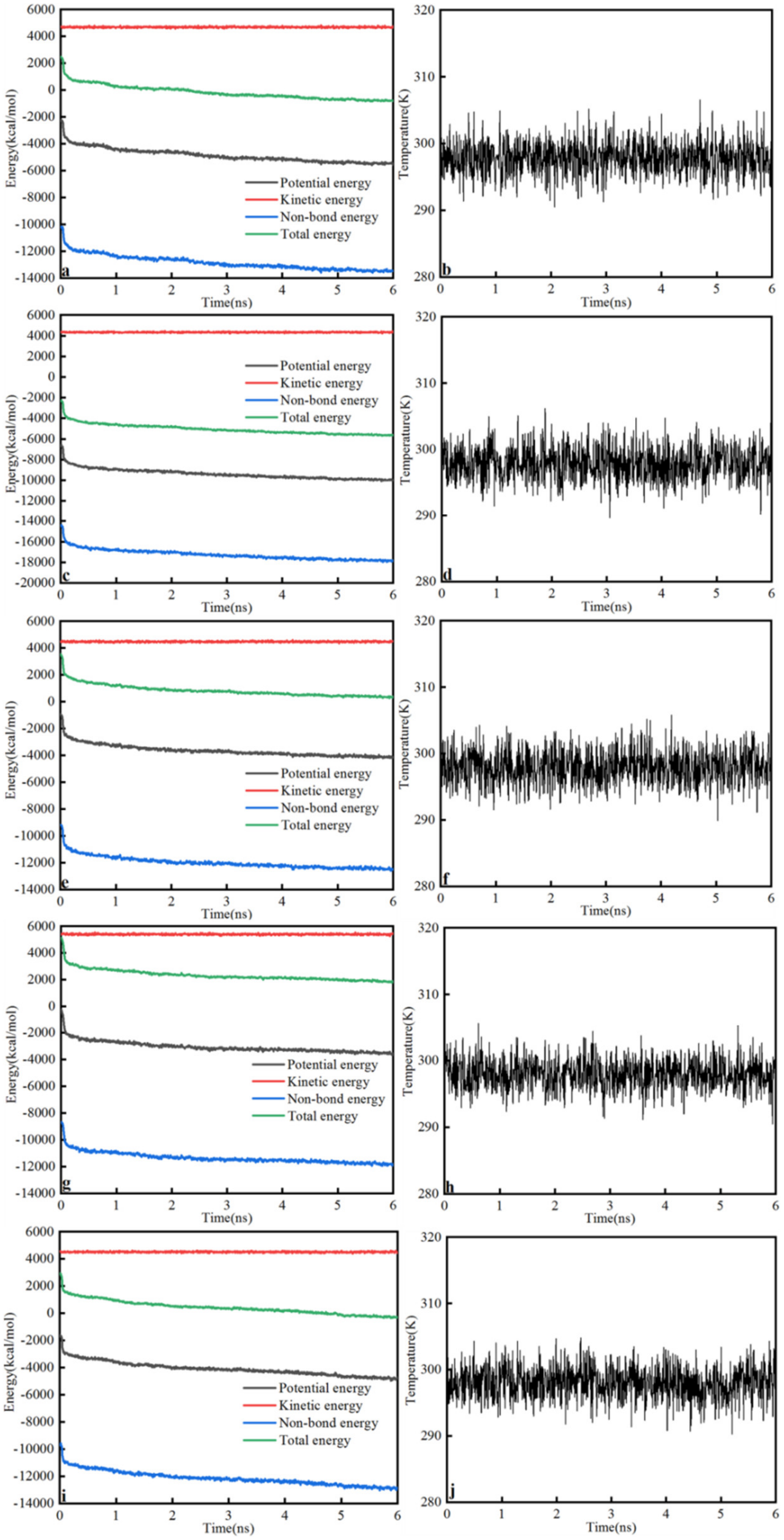

Figure S8. The change of asphaltenes system energy and temperature with time (**a**,**b**) CTAB; (**c**,**d**) SDS; (**e**,**f**) TX−100; (**g**,**h**) sophorolipid; (**i**,**j**) Rhamnolipid.
